# Two Main Biosynthesis Pathways Involved in the Synthesis of the Floral Aroma of the Nacional Cocoa Variety

**DOI:** 10.3389/fpls.2021.681979

**Published:** 2021-09-24

**Authors:** Kelly Colonges, Juan-Carlos Jimenez, Alejandra Saltos, Edward Seguine, Rey Gastón Loor Solorzano, Olivier Fouet, Xavier Argout, Sophie Assemat, Fabrice Davrieux, Emile Cros, Renaud Boulanger, Claire Lanaud

**Affiliations:** ^1^Cirad, UMR Amélioration Génétique et Adaptation des Plantes Méditerranéennes et Tropicales, Montpellier, France; ^2^Amélioration Génétique et Adaptation des Plantes Méditerranéennes et Tropicales, Univ Montpellier, Cirad, INRAE, Institut Agro, Montpellier, France; ^3^Cirad, UMR Qualisud, Montpellier, France; ^4^Qualisud, Univ Montpellier, Avignon Université, Cirad, Institut Agro, IRD, Université de La Réunion, Montpellier, France; ^5^Cocoa and Coffee Research Program, Instituto Nacional de Investigacion Agropecurias, Quito, Ecuador; ^6^Guittard, Burlingame, CA, United States

**Keywords:** GWAS, cocoa aroma, floral, monoterpenes, phenolic compounds

## Abstract

*Theobroma cacao* is the only source that allows the production of chocolate. It is of major economic importance for producing countries such as Ecuador, which is the third-largest cocoa producer in the world. Cocoa is classified into two groups: bulk cocoa and aromatic fine flavour cocoa. In contrast to bulk cocoa, fine flavour cocoa is characterised by fruity and floral notes. One of the characteristics of Nacional cocoa, the emblematic cocoa of Ecuador, is its aromatic ARRIBA flavour. This aroma is mainly composed of floral notes whose genetic and biochemical origin is not well-known. This research objective is to study the genetic and biochemical determinism of the floral aroma of modern Nacional cocoa variety from Ecuador. Genome-Wide Association Study (GWAS) was conducted on a population of 152 genotypes of cocoa trees belonging to the population variety of modern Nacional. Genome-Wide Association Study was conducted by combining SSR and SNP genotyping, assaying biochemical compounds (in roasted and unroasted beans), and sensory evaluations from various tastings. This analysis highlighted different areas of association for all types of traits. In a second step, a search for candidate genes in these association zones was undertaken, which made it possible to find genes potentially involved in the biosynthesis pathway of the biochemical compound identified in associations. Our results show that two biosynthesis pathways seem to be mainly related to the floral note of Nacional cocoa: the monoterpene biosynthesis pathway and the L-phenylalanine degradation pathway. As already suggested, the genetic background would therefore appear as largely explaining the floral note of cocoa.

## Introduction

*Theobroma cacao* L. is native to the tropical rainforests of northern South America and is a member of the family *Malvaceae* (Bayer and Kubitzki, 2003). The cocoa tree is a diploid (2*n* = 20) with a small genome that is now sequenced and of which 96.7% of the assembly is anchored on all 10 chromosomes (Argout et al., 2011, 2017; Motamayor et al., 2013).

Cocoa farming represents an important economic issue for many tropical countries because it is the only source of chocolate supply. In 2018/2019, cocoa production represented more than 4,780 thousand tonnes worldwide. The three largest producers are Ivory Coast, Ghana, and Ecuador with, respectively, 1,964, 905, and 287 thousand tonnes produced (ICCO, 2020). Even if Africa remains the leading producer, America maintains its reputation thanks to the aromatic quality of its cocoa. Cocoa is classified into two types of products: bulk cocoa and fine flavour cocoa. Fine flavour cocoa is characterised by fruity and floral notes unlike bulk cocoa (Sukha et al., 2008). Bulk cocoa accounts for around 95% of world production compared to 5% for fine flavour cocoa. *Theobroma* cacao L. is highly diverse and has been classified into 10 genetic groups: Amelonado, Contamana, Criollo, Curaray, Guiana, Iquitos, Marañón, Nanay, Nacional, and Purùs (Motamayor et al., 2008).

Nowadays, three varieties are mainly capable to produce fine flavour cocoa: Criollo, Nacional, and Trinitario (hybrids between Criollo and Amelonado). Criollo is not widely cultivated because of its high susceptibility to diseases and low vigour (Cheesman, 1944). Nacional is native to Ecuador and is well-known for its Arriba floral flavour. It is for this reason that it is sought after by chocolate makers. It is characterised by floral and woody notes (Luna et al., 2002). Also, Nacional is known for its low astringency and bitterness (International Cocoa Organization, 2017). The first hypothesis explaining floral notes of Arriba flavour was suggested by Ziegleder (1990) who observed that linalool, a volatile compound (VOC) belonging to monoterpenes, was observed in higher concentration in Nacional cocoa.

Overall, fine flavours are often produced during the fermentation process (Rodriguez-Campos et al., 2011). The cocoa fermentation takes place in two stages: first, the alcoholic fermentation made by yeast thanks to the presence of sugar in the cocoa pulp, then, there is an acetic fermentation carried out by bacteria (Ho et al., 2014). Fermentations produce aroma precursors but also VOCs. An adaptation of fermentation conditions is required to improve cocoa beans fine flavour. Fermentation time has an important effect on the concentration of different VOCs, as for some alcohol concentrations, which decreases from 2 to 8 days of fermentation (Rodriguez-Campos et al., 2012; Hamdouche et al., 2019). The drying process occurs after fermentation, which allows stopping it. This step is very important for cocoa bean conservation. It allows moisture decrease from 80 to under 8% (Cros and Jeanjean, 1995; Afoakwa et al., 2008). The artificial drying temperature can also influence the aromatic fraction with a decrease in isobutyric acid and an increase in tri and tetramethylpyrazine at lower drying temperature (70 vs. 80°C) (Rodriguez-Campos et al., 2012).

Cocoa beans have been studied to understand how their specific flavour is synthesised. A study on unfermented dry cocoa beans showed that terpenes are already present and important for fruity and floral aromas, even without fermentation (Qin et al., 2017). Other scientists have also proven the importance of terpenes such as linalool or epoxylinalool in cocoa fine flavour after fermentation (Kadow et al., 2013; Cevallos-Cevallos et al., 2018). Kadow et al. (2013) demonstrated that the aroma specificity depends on the presence of VOCs and can be different depending on the genotype. The most important VOCs for the floral aroma of cocoa have been identified: they include terpenes mainly linalool, 2-phenylethanol (or phenylethyl alcohol), 2-phenylethyl acetate, and acetophenone (Ziegleder, 1990, 2009; Afoakwa et al., 2008; Kadow et al., 2013; Cevallos-Cevallos et al., 2018; Utrilla-Vázquez et al., 2020). Rottiers et al. (2019) also compared the compounds contained in cocoa beans from the modern Nacional (EET varieties) and a standard cocoa variety CCN51. They were able to identify 14 compounds known to have a floral taste by GC-MS. Only five of them were found during the analysis with an electronic nose: 2-phenylacetaldehyde, 2-phenylethyl acetate, 2-phenylethanol, acetophenone, and linalool. However, other VOCs could be responsible for floral aroma (Schwab et al., 2008).

The biosynthesis pathway of aromatic compounds has been studied. Linalool is a volatile floral compound present in various flowers as *Clarkia breweri*, rose, Chinese jasmine green tea, and wine (Dudareva et al., 1996; Ito et al., 2002; Genovese et al., 2007; Feng et al., 2014). Its biosynthesis pathway is very well-studied. Pichersky et al. (1994) highlighted the linalool biosynthesis pathway in *C. breweri* flowers. They observed the transformation of geranyl pyrophosphate (GPP) to linalool by linalool synthase (LIS). Subsequently, the linalool was transformed into 6,7-epoxylinalool. The 6,7-epoxylialool was then converted to pyranoid linalool oxide or furanoid linalool oxide. Other studies showed that cytochrome P450 is responsible for the transformation of linalool to 6,7-epoxylinalool and cyclases for the transformation of 6,7-epoxylinalool to pyranoid linalool oxide or furanoid linalool oxide (Kreck et al., 2003; Meesters et al., 2007; Chen et al., 2010).

2-phenylethanol (or phenylethyl alcohol) has been found in muscadine grape juice, wine, and roses (Baek et al., 1997; Helsper et al., 1998; Genovese et al., 2007). 2-phenetylethanol and 2-phenylethyl acetate were observed in the same biosynthesis pathway in roses (Roccia et al., 2019). L-phenylalanine is converted to 2-phenylacetaldehyde by phenyl acetaldehyde synthase (PAAS). Subsequently, 2-phenylacetaldehyde is reduced to 2-phenylethanol by phenyl acetaldehyde reductase (PAR). Next, 2-phenylethanol is acetylated to 2-phenylethyl acetate by acetyl-coenzyme a: geraniol/citronellol acetyl transferase (AAT) (Roccia et al., 2019).

Acetophenone has been found in muscadine grape juice and Camellia (Baek et al., 1997; Dong et al., 2012). It has the same precursor as 2-phenylethanol but has a parallel biosynthesis pathway identified in the fungus *Bjerkandera adusta*. The transformation of L-phenylalanine to 2-phenylethanol is due to the non-oxidative degradation pathway of L-phenylalanine, while L-phenylalanine transformation to acetophenone belongs to β-oxidation pathway (Lapadatescu et al., 2000). In Camellia, the acetophenone biosynthesis pathway has been characterised (Dong et al., 2012). First, L-phenylalanine (L-phe) is converted to cinnamic acid (CA). Next, CA is transformed into 3-hydroxy-3-phenylpropionic acid (HPPA). 3-Hydroxy-3-phenylpropionic acid is converted to 3-phenylpropionic acid (PPA) and PPA is transformed into acetophenone. The enzymes involved in these reactions have not yet been identified.

Few studies were carried out on the genetic determinants of cocoa qualities. The first were based on QTL analyses of some sensory traits and fat content (Lanaud et al., 2003) and also showed hotspots of VOCs co-located on the genome (Lanaud et al., 2012).

This study aims to contribute to the deciphering of the genetic and biochemical determinism of Nacional cocoa floral notes. To this end, we conducted a genome-wide association study (GWAS) on a modern cultivated Nacional population, composed of trees resulting from hybridizations between three contrasting main ancestors: Criollo, Amelonado, and the ancestral Nacional variety. This population was characterised by VOCs and sensory analyses and presented a high degree of variability. Thanks to the availability of the genome sequence and high-density SNP genotyping, candidate genes involved in key traits could be proposed.

## Materials and Methods

### Vegetal Material

The plant material used for these experiments was composed of a collection of 152 cocoa trees from Ecuador conserved in the Pichilingue experimental station of the “Instituto Nacional de Investigaciones Agropecurias” (INIAP) and the “Colecion de Cacao de Aroma Tenguel” (CCAT) of Tenguel. This population represents the Nacional variety currently grown in Ecuador and has been described by Loor (2007).

### Fermentation Processes

Micro-fermentations of cocoa beans were carried out in a wooden box in the most homogeneous way possible with a homogeneous cocoa mass. The process lasted 4 days with two turns at 24 and 72 h after the beginning of the fermentation. Each clone sample (152) was placed in a protective laundry bag and micro-fermented in a cocoa mass. After fermentation, the samples were put in a dry place. They were considered dried when their moisture content was below 8%.

### Sensorial Analysis

One hundred and forty-six individuals were characterised by sensory analyses based on blind tastings carried out on three repetitions per sample. The tastings were carried out on cocoa liquor. The cocoa liquor corresponds to merchant cocoa (dried fermented beans) which have been roasted and crushed. Sixteen floral notes were judged with a score ranging from zero (no floral notes detected) to 10. We used the average of the three replicates for the phenotype of the GWAS analysis (ISCQF, 2020).

This study was managed by Mr. Edward Seguine, whose work consists of conducting sensory analyses of chocolate samples (see attached documents). This study does not require the approval of an ethics committee.

### Volatile Compound Analysis by GC-MS

#### Preparation of Cocoa Samples

The analysis of VOCs was carried out on dried fermented beans and roasted beans. For each sample, 50 g of beans were taken. The beans were deshelled and crushed to obtain nibs. Then, nibs were put in liquid nitrogen and ground with a blender (SEB, France), to obtain cocoa powder, which was stored at −80°C until analysis. In a 10 ml vial, 2.85 g of powder, 1 ml of standard internal solution (butan-1-ol at a concentration of about 600 μg/ml), and 2 ml of distilled water were added.

#### Compounds Extraction

The VOCs of cocoa samples were extracted using the technique of solid-phase microextraction in the headspace (SPME-HS) using a 50/30-μm divinylbenzene/carboxene/polydimethylsiloxane (DVB/CAR/PDMS) fibre provided by Supelco to extract volatiles. The fibre was previously conditioned at 250°C for 3 min and then exposed to the sample headspace at 50°C for 45 min. Extracted aroma VOCs were analysed using an Agilent 6890 N gas chromatography–mass spectrometer (GC–MS) equipped with a Hewlett Packard capillary column DBWAX, 30 m length × 0.25 mm internal diameter × 0.25 μm film thickness (Palo Alto, CA, USA). The GC oven temperature was initially set at 40°C for 5 min, increased to 140°C at a rate of 2°C/min and then increased at a rate of 10 to 250°C for 66 min. The carrier gas was high-purity helium at 1 ml min^−1^. Injection mode was split less at 250°C for 2 min. The selective mass detector was a quadrupole (Hewlett Packard, Model 5973), with an electronic impact ionisation system at 70 eV and at 230°C (Assi-Clair et al., 2019).

#### Compounds Identification

The identification was done by comparing the mass spectra with the commercial NIST Wiley 275L database. No deconvolution was applied. Co-eluted VOCs were excluded from this study, with the exception of cis-ocimene co-eluted with ethyl hexanoate (cis-ocimene + ethyl hexanoate) which showed interesting results.

### DNA Extraction Protocol

DNA extraction was conducted according to Risterucci et al. (2000) protocol.

### Genotyping by SSR

This population was genotyped using SSR markers by Loor (2007). SSR loci were scored individually and alleles were recorded by the presence of polymorphic DNA fragments (alleles) among the individuals of each population. Only those alleles that showed consistent amplification were used in the analysis of results and smeared or weak bands were ignored.

### Genotyping by Sequencing

DNA samples were genotyped by sequencing (GBS) using DArTseq (Diversity Arrays Technology Sequencing) technology (Kilian et al., 2012). This method is based on enzymatic restriction of coding regions of the genome by the restriction enzymes: Pst1 and Mse1. The restriction generated many short fragments, with each locus represented more than 10 times. Then, illumina Hiseq2000 machine sequenced all the fragments and the result was analysed. Reads were aligned with the V2 sequence of the Criollo genome (Argout et al., 2017). Reads that have more than one location were discarded. Markers with unknown locations were discarded for analysis. All the markers used are available on http://tropgenedb.cirad.fr/tropgene/JSP/interface.jsp?module=COCOAinthegenotypessectionandtheCocoa-Nacional-aromasub-section.

### Population's Structure Analysis

The phylogenetic tree was generated using DARwin software (Perrier and Jacquemoud-Collet, 2006). The genetic distances were calculated using the Dice coefficient and the Neighbour-Joining method (Dice, 1945; Saitou and Nei, 1987).

### Association Mapping

The graphic representation of the markers along the 10 chromosomes was made with the R package “CMplot” (Yin, 2020). Several analyses of associations with SNP or SSR markers have been performed:

#### SNP GWAS

First, we performed a GWAS analysis with SNP markers associated with biochemical (146 accessions × 5,195 markers) and sensory (144 accessions × 5,195 markers) traits using TASSEL v5.

For all the traits, we used a mixed model (MLM) on the one hand. The MLM was carried out with a structure matrix, determined by running a principal component analyses (PCA integrated with TASSEL v5 software), considered as a fixed effect, and also with a kinship matrix considered as a random effect as covariates to control the false-positive rate. The option of not compressing and re-evaluating the components of variance for each marker was chosen. The kinship matrix using the identity by state (IBS) pairwise method proposed by Tassel v5 was established.

On the other hand, we used a fixed-effect model (GLM) with a structure matrix, determined by running a PCA. The option of 500 permutations was chosen.

For both methods, quantile-quantile plots were used to graphically evaluate the false-positive numbers observed in the selected model, based on deviations from the uniform law. The threshold was determined using the Bonferonni correction formula as proposed by Gao et al. (2008) with the effective number of independent tests (Meff) used as the denominator and calculated by SimpleM R package (Gao et al., 2010). Meff was 2,796, which corresponds to a *P*-value of approximately 1.79e^−05^. The significance of all markers was plotted using Manhattan plots with the R QQman package.

#### SSR GWAS

We performed an analysis with SSR markers associated with biochemical (180 accessions × 180 markers) and sensory (197 accessions × 180 markers) traits using TASSEL v3. We used a fixed-effect model (GLM) with a structure matrix; the option of 500 permutations was chosen. The threshold was determined using the Bonferonni correction corresponding to a p-value about 2.78e^−04^.

The borders of the association zones were calculated using Haploview (Barrett et al., 2005). The haplotypic blocks were calculated with SNP data using Haploview with the association test, Family trio data, Standard TDT, and ignore pairwise comparison of markers above to 10,000 kb calculation parameters. The haplotypic block information was used to determine the confidence intervals of association areas.

The physical maps with the QTL representation were created using SpiderMap v1.7.1 software (Rami, 2007 unpublished). The size of the dots is correlated to the R2.

The identification of candidate genes was performed using the *Theobroma cacao* genome sequence (Argout et al., 2017).

### Statistical Analysis

Principal component analyses analysis and visualisation were made with the “mixOmics” R package. Calculation of correlation was made with “agricolae” R package and visualisation of correlation matrix with “corrplot” R package.

## Results

### Genetic Diversity and Population Structure

**Figure 1:**
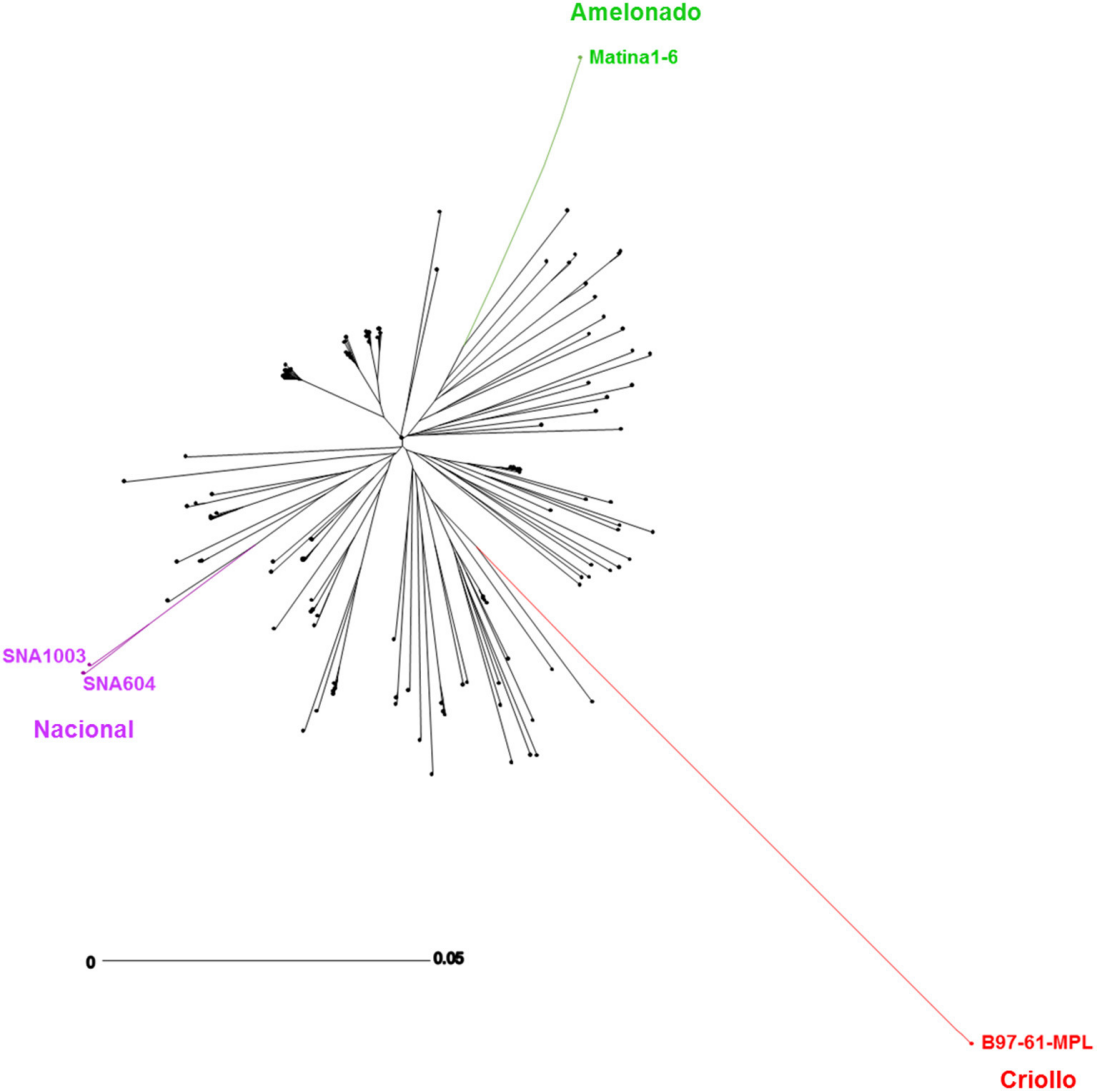
Phylogenetic tree representing the modern Nacional population and its ancestors. Phylogenetic tree of the individuals of the studied population made with 4,130 SNPs and including the ancestor controls of the population: in red, the Criollo variety (B97-61-B2); in purple, the Nacional variety (SNA604, SNA1003); in green, the Amelonado variety (Matina 1–6); in black, the individuals of the studied population. The graph's scale represents the edge lengths which are proportional to the genetic distance.

The population studied represents the modern population of the Nacional variety cultivated in Ecuador. It is the result of various crosses between three main ancestors: the Criollo, the Amelonado, and the ancient Nacional varieties (Loor, 2007). Using SNP markers, the structure of the genetic diversity of the population was studied. There was a continuous distribution of population trees between the three ancestors (Criollo, Nacional, and Amelonado varieties) as shown in Figure 1. Loor (2007) had also shown this distribution using microsatellite markers.

### Characterisation of the Studied Traits

To identify the areas of *T. cacao* genome involved in the synthesis of typical Nacional floral aromas, a GWAS was conducted with two types of traits: the VOCs present in cocoa beans (before and after roasting) and sensory analysis data.

#### Sensorial Traits Analysis

Sixteen floral notes were determined by sensory analyses performed on cocoa liquor. A total of 16 sensorial traits were therefore used for this study (Supplementary Table 1).

**Figure 2:**
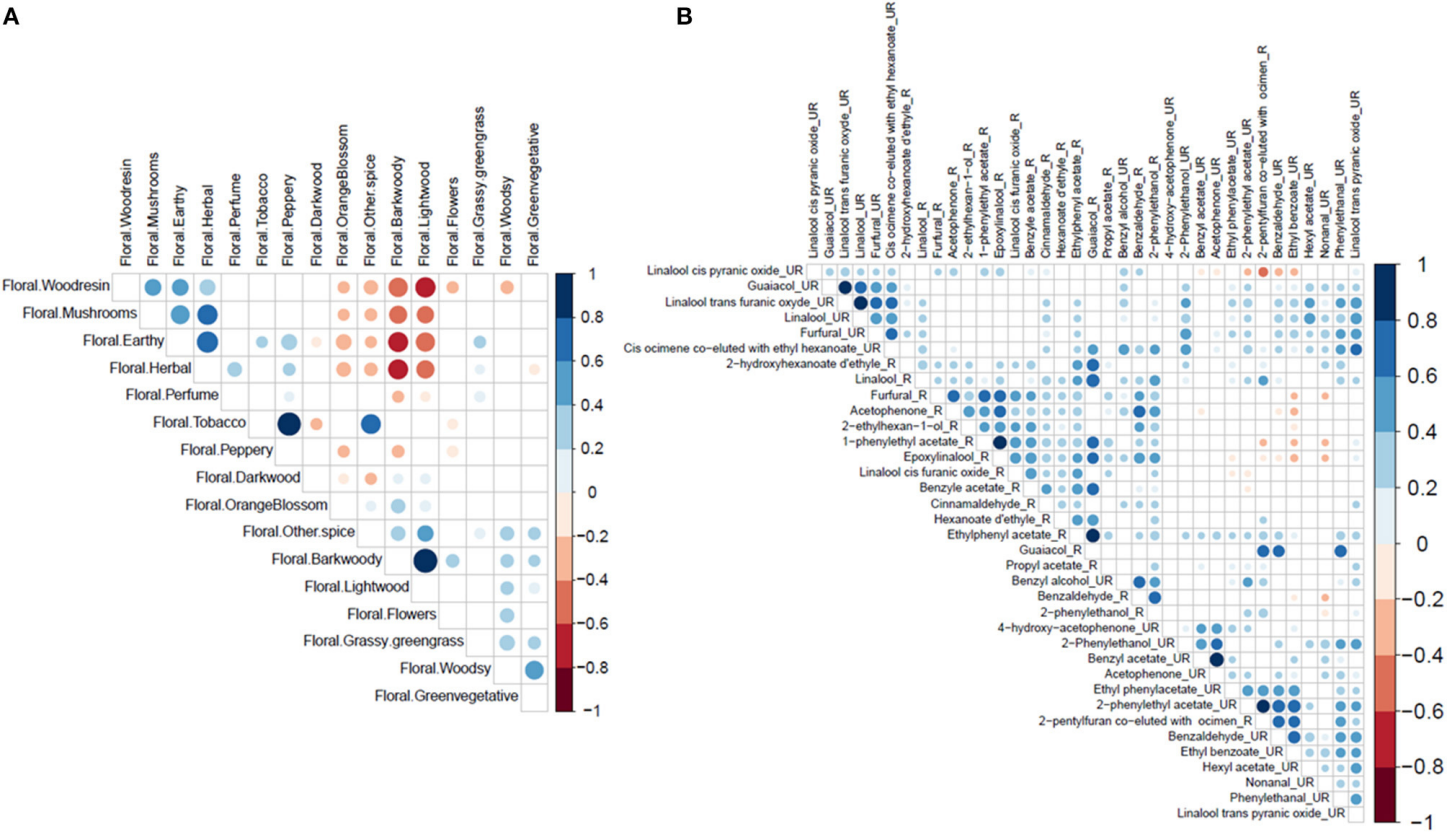
Significant correlation matrix. **(A)** Correlation matrix between the sensorial profiles determined in cocoa liquor. **(B)** Correlation matrix between the biochemical compounds measured in unroasted (UR) and roasted beans (R). The correlations were calculated by the Pearson method. The white boxes represent no significant correlations. The colour of the circles corresponds to Pearson's correlation coefficient. The areas of circles correspond to a *p*-value of correlation coefficients. The *p*-value threshold for a significant correlation is 0.05. The different shades of blue represent a positive correlation coefficient while the different shades of red represent a negative correlation coefficient. The intensity of the colour depends on the strength of the R2 correlation coefficient. The scale on the right indicates the interpretations of different colours.

Principal component analysis for sensory traits showed continuous variation in the population (Supplementary Figure 1). Axis 1 is mainly defined by the aromatic notes: browned flavour, floral bark woody and smoky. Axis 2 is mainly defined by the aromatic notes: floral tobacco, fruity acidity, and astringency. Correlation analyses between sensory traits showed strong positive and negative correlations (Figure 2A). These strong correlations suggest either that the correlated sensory notes are produced by the same compounds or that an interaction exists between the perceptions of the two sensory traits.

#### Analysis of Aroma Volatile Compounds

The biochemical characterisation was done on unroasted and roasted beans. Among 160 VOCs identified, 26 VOCs are known to have a floral taste or are involved in biosynthetic pathways of known floral compounds (Table 1). Eighteen of them were detected in unroasted beans and 17 in roasted beans such as linalool, acetophenone, or 2-phenylthanol. These VOC were used to conduct a GWAS analysis (Table 1).

**Table 1:** List of biochemical compounds related to floral traits used for the GWAS analysis of unroasted (UR) and roasted (R) beans.

**UR**	**R**	**Aroma volatile compounds**	**Compound family**	**Aroma**
	X	1-Phenylethyl acetate	Ester	Fruity (Garg et al., 2018)
	X	2-Ethylhexan-1-ol	Alcohol	Leafy, rose (Garg et al., 2018)
X	X	2-Phenylethanol	Alcohol	Rose, honey (Jezussek et al., 2002; Genovese et al., 2007)
X		2-Phenylethyl acetate	Ester	Floral, underwood, rose (Guichard et al., 2003; Genovese et al., 2007; Wang et al., 2014)
X		4-Hydroxy-acetophenone	Ketone	
X	X	Acetophenone	Ketone	Acacia honey, floral, and fruity (Genovese et al., 2007; Wang et al., 2014)
X	X	Benzaldehyde	Aldehyde	Bitter, cherry, almond, fruity (Perestrelo et al., 2006; Pham et al., 2008; Wang et al., 2014)
X	X	Benzyl acetate	Ester	Floral, Jasmin (Ito et al., 2002)
X		Benzyl alcohol	Alcohol	Fruity (Ito et al., 2002)
	X	Cinnamaldehyde	Aldehyde	Spicy, cinnamon (Garg et al., 2018)
	X	Epoxylinalool	Terpene	Floral (Arn and Acree, 1998)
	X	Ethyl 2-hydroxyhexanoate	Ester	Floral (Wang et al., 2014)
X		Ethyl benzoate	Ester	Fruity, violet, candy (Ferreira et al., 1997)
	X	Ethyl dodecanoate	Ester	Floral, fruity, leafy (Garg et al., 2018)
	X	Ethyl hexanoate	Ester	Fruity (strawberry, green apple) and floral (Larsen and Poll, 1992; Ferreira et al., 1997; Genovese et al., 2007; Wang et al., 2014)
X	X	Ethylphenyl acetate	Ester	Rose, floral (Perestrelo et al., 2006)
X	X	Furfural	Furan	Incense, fruity, floral, toasted, sweet, and almond (Ferreira et al., 1997; Colahan-Sederstrom and Peterson, 2005; Wang et al., 2014)
X	X	Guaiacol	Aromatic hydrocarbon	Phenolic, floral, smoky, sweet, medicament (Ferreira et al., 1997; Arn and Acree, 1998; Genovese et al., 2007)
X		Hexyl acetate	Ester	Fruity (pear) and floral (Guichard et al., 2003; Wang et al., 2014)
X	X	Linalool	Terpene	Floral, citrus peel, orange flower (Ferreira et al., 1997; Genovese et al., 2007)
	X	Linalool cis furanic oxide	Terpene	Floral, woody (Arn and Acree, 1998)
X		Linalool cis pyranic oxide	Terpene	Fruity, citrus, green (Arn and Acree, 1998; Ito et al., 2002)
X		Linalool trans furanic oxide	Terpene	Citrus, leafy, floral (Arn and Acree, 1998; Ito et al., 2002)
X		Linalool trans pyranic oxide	Terpene	
X		Nonanal	Aldehyde	Orange-like, floral, soapy (Kumazawa and Masuda, 2002; Mahajan et al., 2004; Karagül-Yüceer et al., 2006)
X		Phenylethanal	Aldehyde	Floral, rose, honey (Perestrelo et al., 2006)
	X	Propyl acetate	Ester	Celery, floral, pear, red fruit (Garg et al., 2018)

*UR, unroasted beans; R, roasted beans*.

*X, biochemical compounds find in unroasted or roasted beans*.

Principal component analyses of aroma VOCs was made (Supplementary Figures 2, 3). Axis 1 of the PCA from analyses of biochemical compounds in unroasted beans is mainly defined by the linalool trans furanic oxide, meso-2,3-butan-di-yl diacetate, and linalool trans pyranic oxide. Axis 2 is mainly defined by ethyl acetate, ethyl-(2-methyl)-propionate, and benzaldehyde. Axis 1 of the PCA from analyses of biochemical compounds in roasted beans is mainly defined by epoxylinalool, 2-acetylpyrrole, and ethylphenyl acetate. Axis 2 is mainly defined by pentan-2-ol, pentan-2-one, and 1.2.5-trimethylbenzene. As with sensory traits, PCA of aroma VOCs showed that the distribution of traits showed a continuous variation within the population which can be explained by the great genetic diversity present in this group of individuals deriving from several generations of crosses.

Correlation analyses between the different traits showed positive correlations between several biochemical compounds in roasted and unroasted beans (Figure 2B). The highest correlations (>0.8) were observed in unroasted beans: between benzyl acetate and acetophenone; between 2-phenylethyl acetate and 2-pentylfuran co-eluted with ocimene; between guaiacol and trans furanic oxide linalool; between trans furanic oxide linalool and linalool. High correlations were also observed in roasted beans: between 1-phenylethyl acetate and epoxylinalool; between ethylphenyl acetate and guaiacol. A negative correlation between −0.4 and −0.6 was observed between linalool cis pyranic oxide and 2-pentylfuran co-eluted with ocimene in unroasted beans.

These various correlations between compounds can be partly explained by the fact that they belong to the same biosynthesis pathway. This is the case for the different terpenes which are strongly correlated or compounds resulting from the degradation of L-phenylalanine (acetophenone, 2-phenylethanol, and benzaldehyde). On the other hand, no strong correlation between biochemical and sensory traits was detected (Supplementary Figure 4).

### Genome-Wide Association Study

The linkage disequilibrium observed in this population amounts to 15 cM (Loor, 2007). Genome-wide association study analyses were performed by different methods (GLM and MLM) and with different types of markers (SSR and SNP).

#### Marker Sorting

To limit the biases due to rare alleles, sorting by the frequencies of the minor alleles (MAF) was done at 5% (MAF5). The population being very heterozygous, the sorting by MAF allowed to eliminate the alleles with a total frequency lower than 5% but left homozygous genotypes very poorly represented (one individual per class). The hypothesis was that the low representation of genotypic classes could induce a bias in the analyses, in the same way as a minor allele. It was therefore undertaken to do a further sorting of markers by discarding markers for which genotype classes had <5% representation of the total population (Minor genotype frequencies, MGF). We conserved markers that had at least seven individuals per genotype class (G7). Several tests were performed such as the comparison of Q–Q plots or the comparison of *p*-values (Zhang et al., 2019) to determine which of the two sorting methods had the least bias (Supplementary Figure 5). None of the tests could determine which of the two was the most biassed. The results differed in some respects, so both marker sorting methods were retained for the GWAS studies.

#### SNP Marker Distribution

**Figure 3:**
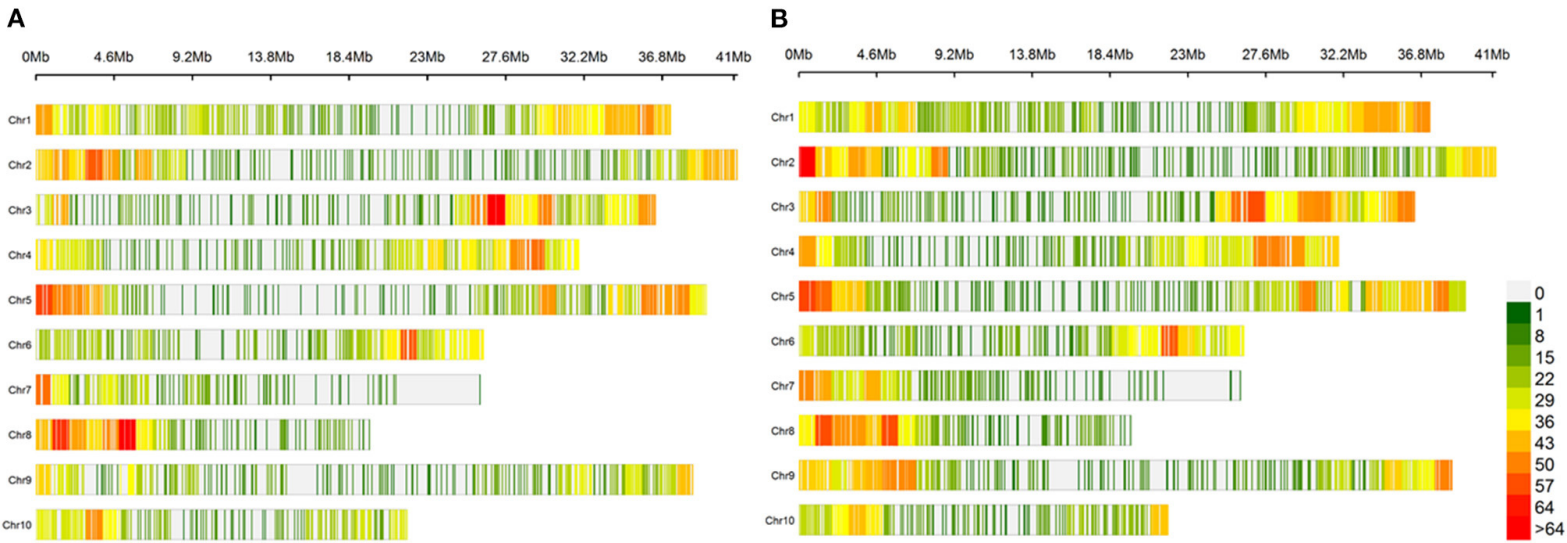
Distribution of markers along the 10 chromosomes of *T. cacao*. **(A)** Distribution of markers from the G7 dataset along the 10 chromosomes of *T. cacao*. The graph shows the distribution of markers along the 10 chromosomes. The density is calculated on a 1 Mb window. The areas without markers are shown in grey. The weakly marked areas are in green and the strongly marked areas are in red. A colour gradient between green and red represents the marking gradient. **(B)** Distribution of markers from the MAF5 dataset along the 10 chromosomes of *T. cacao*.

For the GWAS, SNPs were selected without missing data and with a genotype frequency above 5% or a MAF above 5%. The final data set consisted of 5,195 SNP markers for the G7 data set and 6,541 SNP markers for the MAF5 data set (Ruiz et al., 2017). The SNP markers are well spread over all 10 chromosomes of *T. cocoa*. However, a decrease in marker density is observed in the centromeric and peri-centromeric areas (Figure 3).

#### Determination of Confidence Intervals of Associations Based on Haplotypes

Haplotypes were calculated based on the known linkage disequilibrium of the population which is 15 cM, corresponding to 10,000 kb. A total of 681 haplotypic blocks were thus determined with a minimum of 42 haplotypic blocks present on chromosome 8 and a maximum of 96 haplotypic blocks present on chromosome 1. Confidence intervals were defined based on these haplotypic blocks. In this paper, each association zone, thus corresponding to a haplotypic block, is represented by its association peak. The association peak corresponds to the marker for which the association is the most significant.

#### Comparison of the Four Different Methods Used for SNP Association Studies

The GLM method has made it possible to highlight more areas of association than the MLM method. In both cases, the use of the set of markers sorted according to a 5% MAF (MAF5) also made it possible to highlight more association zones: 333 against 295 for the GLM method and 152 against 94 for the MLM method. The MLM method, therefore, appears to be more stringent.

**Figure 4:**
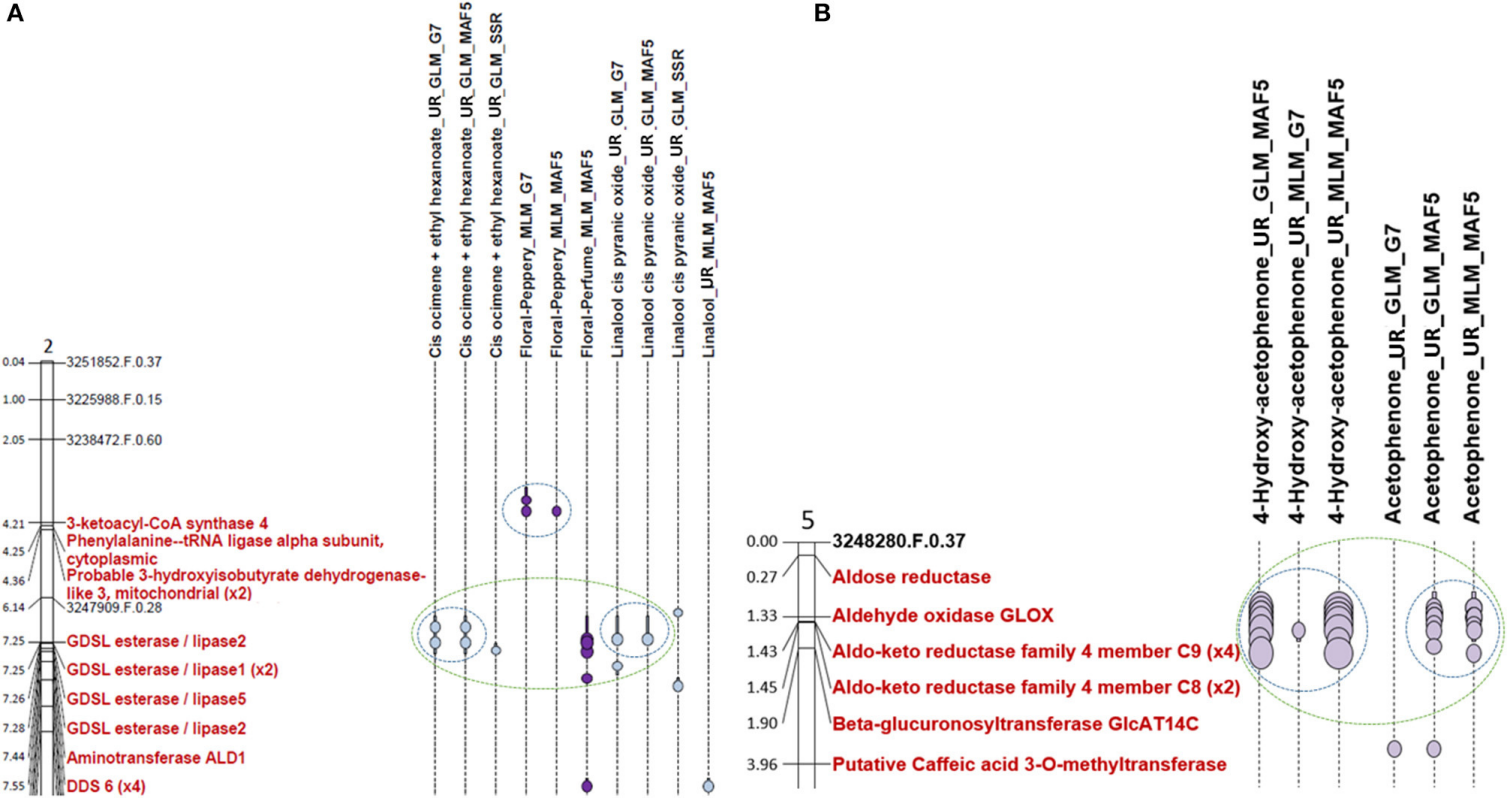
Extract of chromosome 2 map and chromosome 5 map. **(A)** Extract from the chromosome 2 map representing the associations detected for compounds involved in the monoterpene biosynthetic pathway. **(B)** Extract from the chromosome 5 map representing the associations detected for compounds involved in the L-phenylalanine degradation pathway. The light blue dots represent the peaks of associations in relation to traits whose beans have not been roasted. The dark purple dots represent the peaks of association in relation to traits whose beans have been roasted. The bars around these points correspond to the confidence intervals of the association zone. Co-locations are represented by a blue circle for the associations co-localised for the same trait and identified by different methods. Co-locations are represented with a green circle for the co-locations between different biochemical compounds. Candidate genes are written in red. One scale unit on the chromosome corresponds to 1 Mb.

Some areas of the association are common for different methods. For example, in the case of terpene relatives' traits, 63 co-locations between positive associations for different methods for the same trait was found on all chromosomes except chromosome 4 and 8. A co-localisation between GLM_MAF5 and GLM_G7 methods for linalool cis pyranic oxide (UR) was observed on chromosome 2 as shown in Figure 4A. In the case of L-phenylalanine relatives' traits, co-locations of the association zones between the different methods for the same trait was observed on all chromosomes. This is the case for example on chromosome 5 where co-localisation of associations for GLM_MAF5, MLM_G7, and MLM_MAF5 linked to 4-hydroxyacetophenone (UR) was observed (Supplementary Figure 6).

### Identification of Significant Associations for Sensorial Traits

Among all the associations, only 38 are related to the sensory data with floral notes. Out of a total of 16 floral perceptions, significant associations were detected for 11 of them, on all chromosomes except chromosome 5 and chromosome 7. Only one area of association was revealed for each of the six floral notes: the floral notes bark woody, dark wood, mushrooms, orange blossom, other spice, and tobacco (Table 2). Four association zones were also detected for the floral note Lightwood on chromosome 1. The area of strongest association detected for the light wood floral note and the tobacco floral note is in the same haplotypic block. The floral note that allowed detecting the most areas of association is the floral perfume where 13 areas were highlighted. The variation in the floral perfume note is the one that seems to be the most explained by the genetic variation observed, with an explanation rate for variation in the trait of 24%.

**Table 2:** Most significant association detected for each of the sensory floral traits.

**CH**	**Position of the association peak (bp)**	**N° hap. bloc**	**Floral note detected**	***p-value* of the strongest association**	**Explanation rate of the trait of the strongest association (%)**	**Total number of associations for the character**
1	4,079,457	NA	Floral-other spice	1,45E-05	14	1
1	4,129,759	10	Floral-lightwood	2,56E-06	16	4
1	4,131,970	10	Floral-tobacco	4,52E-06	16	1
2	3,606,270	NA	Floral-peppery	9,42E-07	15	5
2	7,476,546	36	Floral-perfume	1,87E-09	24	13
6	21,137,437	45	Floral-wood resin	8,74E-07	15	6
6	26,160,073	NA	Floral-dark wood	5,78E-06	13	1
8	15,196,137	37	Floral-orange blossom	0,000132	12	1
9	38,188,583	58	Floral-mushrooms	1,57E-08	22	1
9	4,248,470	17	Floral-bark woody	6,56E-06	15	1
9	6,245,108	21	Floral-green vegetative	7,45E-09	23	4

*CH, chromosome; hap, haplotypic; bp, base pair*.

### Identification of Significant Associations for Aroma Volatile Compounds

The GWAS analyses brought to light 393 association zones. Some of them were detected with several VOCs. All the associations found can be consulted in the Supplementary Table 2.

Significant associations for 18 VOCs in unroasted beans and 17 volatile compounds in roasted beans were identified (Supplementary Table 2). No association zones were detected for five VOCs, four of which were assayed in roasted beans: ethylphenyl acetate (UR), ethyl 2-hydroxyhexanoate (R), ethyl hexanoate (R), guaiacol (R), and cis linalool oxide (R).

Two major pathways for the biosynthesis of compounds known to have a floral taste, among those compounds for which a significant association was detected, seem to be particularly represented: the monoterpene biosynthesis pathway and, the L-phenylalanine degradation pathway that allows the synthesis of, among others, acetophenone and 2-phenylethanol.

The results obtained were mapped to visualise the areas of significant associations, their locations, as well as possible co-locations between them. Two maps were made. A map with the results of significant associations related to the compounds involved in the terpene biosynthesis pathway and the floral traits from the sensorial evaluation. A second map includes the results of the significant associations of floral tastes and of compounds involved in the degradation pathway of L-phenylalanine which allows, the synthesis of acetophenone and 2-phenylethanol known to have a floral taste. Some results differ between the different methods (GLM and MLM) or the sorting of SNP markers (MAF5 or G7) or between the type of SNP and SSR markers. All results are shown on the maps in Supplementary Figures 6, 7. Results that are repeatable between methods appear to be the most conclusive.

### Significant Associations Identified for the Biochemical Compounds Involved in Terpene Biosynthetic Pathway

**Figure 5:**
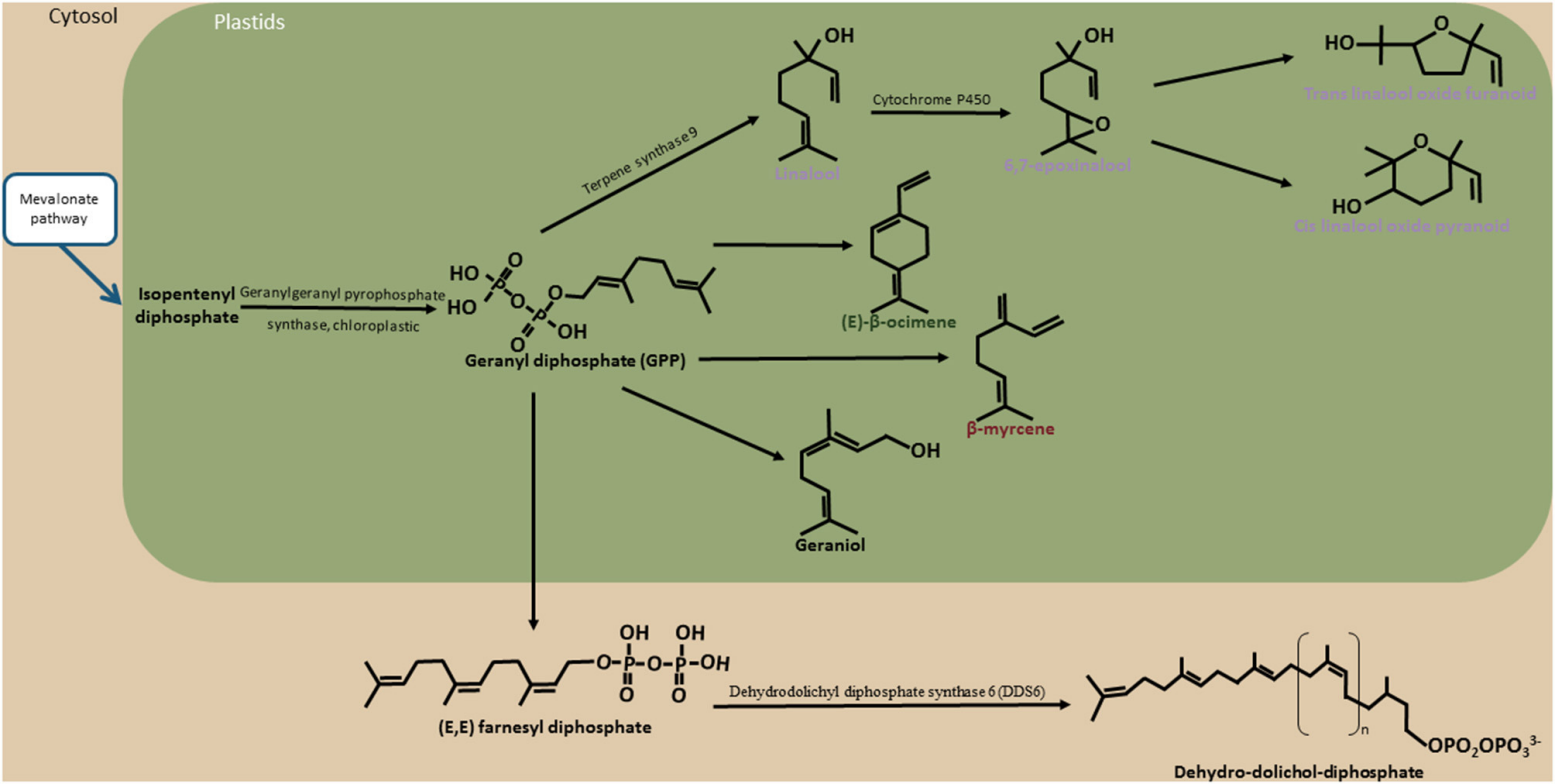
Terpene biosynthesis pathway. The schema illustrates the different biosynthesis pathways of compounds belonging to the terpene biosynthesis pathway identified in cocoa. Compounds known to have a floral taste are noted in purple. The blue arrows represent the bridges between the terpene biosynthesis pathway and mevalonate pathways. The names of these other biosynthetic pathways are framed in blue. The black arrows represent the enzymatic actions. The names of the enzymes are indicated (when identified) around these arrows. In green represented the limit of the plastids. In light brown are represented the limits of cytosol.

Among the 27 compounds related to the floral note, six VOCs derived from the terpene biosynthesis pathway: linalool (UR and R), trans furanic oxide linalool (UR), cis pyranic oxide linalool (UR), epoxylinalool (R), and cis ocimene co-eluted with ethyl hexanoate (UR) (Figure 5). Eighteen zones of association were revealed for the linalool in unroasted beans (UR) against two zones for linalool in roasted beans (R). The most significant association of linalool (UR) was found on chromosome 7 while that of linalool (R) was found on chromosome 6. Twenty-nine association zones were highlighted for the linalool trans furanic oxide (UR). The most significant association linked to linalool trans furanic oxide (UR) was detected on chromosome 7 which is in the same haplotypic bloc of the most significant association of cis ocimene co-eluted with ethyl hexanoate (UR). Twenty-seven associations were observed for linalool cis pyranic oxide (UR). Finally, thirty-eight areas of associations were revealed for the epoxylinalool (R) (Table 3; Supplementary Table 2).

**Table 3:** Most significant associations for biochemical compounds related to terpene pathway.

**CH**	**Position of the association peak (bp)**	**N° hap. bloc**	**Traits**	***p-value* of the strongest association**	**Explanation rate of the trait of the strongest association (%)**	**Associations detected**
1	6,448,063	23	Epoxylinalool_R ^*^	3,23E-11	32	38
6	5,543,124	17	Linalool_ R ^*^	5,57E-06	16	2
7	5,607,833	24	Linalool trans furanic oxyde_UR ^*^	5,51E-10	29	29
7	5,607,833	24	Cis ocimene + ethyl hexanoate_UR	3,73E-12	35	42
7	10,459,413	31	Linalool_ UR ^*^	3,53E-13	37	18
10	6,167,221	27	Linalool cis pyranic oxide_ UR ^*^	3,27E-07	23	27

*The most significant association detected for each compound is reported. CH, chromosome; hap., haplotypic; UR, unroasted beans; R, roasted beans*.

* *Biochemical compounds known for floral notes, bp, base pair*.

The map with the results for terpenes (Supplementary Figure 7) shows several interesting results. Among a large number of associations, several co-locations can be observed between different biochemical compounds involved in the terpene pathway. For example, a co-localisation between the Linalool (UR), the Linalool cis-pyranic oxide (UR), and the Linalool trans-furanic oxide (UR) was observed in chromosome 6 (Supplementary Figure 7). This suggests the greater likelihood that most of these compounds already known for their floral notes are well-involved in floral notes of Nacional cocoa.

#### Co-locations Between Biochemical Compounds

Sixteen co-locations between different biochemical compounds were also observed on chromosomes 2, 4, 5, 7, 9, and 10, for example on chromosome 2 between the linalool cis pyranic oxide (UR) and cis-ocimene co-eluted with ethyl hexanoate (Figure 4A). Various numbers of co-locations could be observed according to chromosomes. Only one co-location are observed on chromosome 9 and chromosome 10 and five co-locations were highlighted on chromosome 7 (Supplementary Figure 7). Co-localisations between association zones identified for different VOCs can be explained by their belonging to the same biosynthesis pathway such as for linalool trans furanic oxide (UR) and linalool (UR) on chromosome 3, or for cis pyranic oxide (UR) and epoxylinalool (R) on chromosome 4 (Supplementary Figure 7). It can then be thought that this zone of associations is due to the presence of a gene coding for an enzyme that is part of this biosynthetic pathway. To verify this hypothesis, we have begun to search for candidate genes at the level of the association zones.

#### Co-locations Between Biochemical Compounds and Sensorial Traits

Seven co-locations between at least one biochemical compound and a floral note were detected on chromosomes 1 and 2. On chromosome 1, two co-locations were observed between epoxylinalool (R) and the floral note lightwood and one between epoxylinalool (R), floral notes lightwood and floral notes tobacco (Supplementary Figure 7). On chromosome 2, a co-localisation exists between cis ocimene co-eluted with ethyl hexanoate (UR), cis pyranic oxide linalool (UR), and floral scent (Figure 4A). A co-localisation is also observable between cis ocimene co-eluted with ethyl hexanoate (UR) and floral perfume. A co-localisation is also observable between linalool (UR) and the floral perfume note (Supplementary Figure 7).

### Significant Associations Identified for the Biochemical Compounds Involved in the Degradation of L-Phenylalanine Pathway

**Figure 6:**
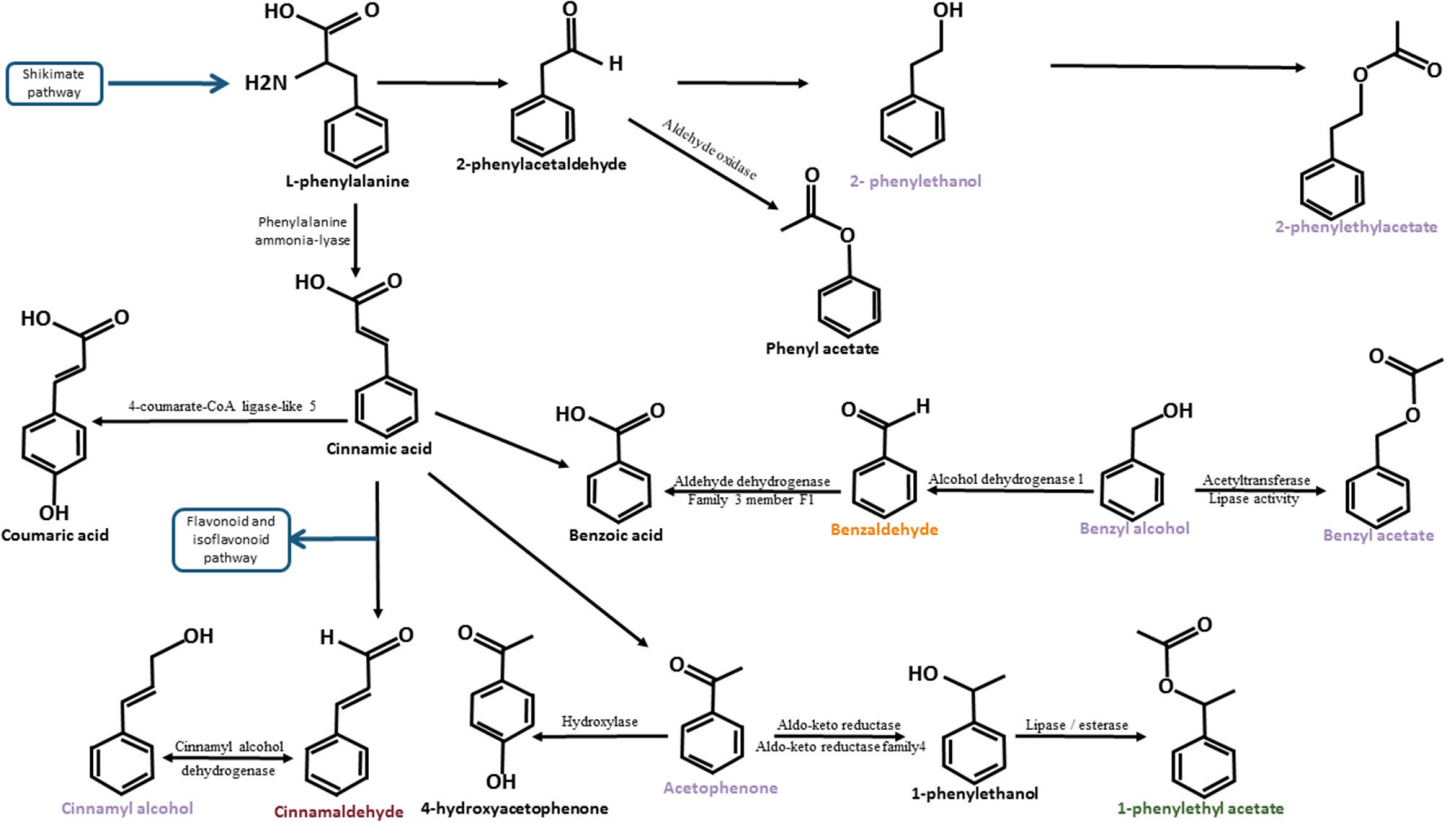
Degradation pathway of L-phenylalanine adapted from Lapadatescu et al. (2000). The schema illustrates the different biosynthesis pathways of compounds belonging to the L-phenylalanine degradation pathway identified in cocoa. Compounds known to have a floral taste are noted in purple. Compounds known to have a fruity taste are noted in orange. Compounds known to have a spicy note are noted in dark red. The blue arrows represent the bridges between the L-phenylalanine degradation pathway and other biosynthetic pathways. The names of these other biosynthetic pathways are framed in blue. Black arrows represent the enzymatic actions. The names of the enzymes are indicated (when identified) around these arrows.

Eighteen compounds for which significant associations have been identified appear to be involved in the degradation pathway of L-phenylalanine to either 2-phenylethanol or acetophenone (Table 4; Figure 6). Among these compounds for two of them, ethylphenyl acetate (R) and phenylethanal (UR), only one zone of the association was identified. The most significant association for phenylethanal (UR) co-localises with the strongest association detected for linalool (R) on chromosome 6. Thirty-six association zones were showed for acetophenone (UR) compared to 40 for acetophenone (R). The most significant association of acetophenone (UR) is on chromosome 2 while that of acetophenone (R) is on chromosome 6. Two hundred and six association zones were detected for cinnamaldehyde (R). Twelve zones of associations were revealed for 2-phenylethanol (UR) and three for 2-phenylethanol (R). The most significant association zones for 2-phenylethanol (UR) and (R) are located on chromosome 4 but at a different position. Two association zones were highlighted for ethyl benzoate (UR). Three areas of association were revealed for 2-phenylethyl acetate (UR). Two zones of associations were revealed for benzaldehyde (UR) against 72 with benzaldehyde (R). Benzaldehyde (UR) presents its most significant association on chromosome 7, while that of benzaldehyde (R) is located on chromosome 6. Thirty-eight association zones were revealed for benzyl acetate (UR) against two for benzyl acetate (R). Twenty-nine association zones were highlighted for 4-hydroxy acetophenone (UR). Seven regions of associations were revealed 2-ethylhexan-1-ol (R). Seventy-three association areas were highlighted for 1-phenylethyl acetate (R). The last two compounds involved in these biosynthetic pathways, benzyl acetate (R) and 1-phenylethyl acetate (R), have their most significant area of association co-locating and forming part of the same haplotypic block number 26 on chromosome 10. The variation of two biochemical compounds seems to be explained mainly by genetic variation. Indeed, the variation in the concentration of 4-hydroxy-acetophenone is explained at 79% by the strongest association zone as well as the variation in cinnamaldehyde which is explained at 65% by the association zone.

**Table 4:** Most significant associations for biochemical compounds related to L-phenylalanine degradation pathway.

**CH**	**Position of the association peak (bp)**	**N° hap. bloc**	**Traits**	***p-value of the strongest association***	**Explanation rate of the trait of the strongest association (%)**	**Associations detected**
10	5,308,832	26	1-Phenylethyl acetate_ R	1,17E-10	31	73
9	1,099,704	5	2-Ethylhexan-1-ol_ R ^*^	2,61E-08	29	7
4	23,646,147	40	2-Phenylethanol_ R ^*^	2,12E-06	16	3
4	17,349,904	NA	2-Phenylethanol_ UR ^*^	9,89E-07	18	12
5	29,926,884	53	2-Phenylethyl acetate_ UR ^*^	6,92E-06	18	3
7	2,815,797	11	4-Hydroxy-acetophenone_ UR	4,86E-43	79	29
6	23,097,785	58	Acetophenone_ R ^*^	1,59E-08	25	40
2	8,389,914	39	Acetophenone_ UR ^*^	2,53E-12	31	36
6	25,213,164	65	Benzaldehyde_ R	8,53E-09	26	72
7	10,459,413	31	Benzaldehyde_ UR	1,32E-05	17	2
7	2,092,063	9	Benzyl acetate_ UR ^*^	2,36E-15	41	38
10	5,228,191	26	Benzyl acetate_ R ^*^	8,15E-06	18	2
2	7,448,797	36	Benzylic alcohol_ UR	2,12E-08	25	42
3	31,503,427	58	Cinnamaldehyde_ R	1,39E-28	65	206
5	27,513,744	45	Ethyl benzoate_ UR ^*^	2,33E-06	18	2
1	36,447,062	91	Ethylphenyl acetate_ R ^*^	1,35E-05	17	1
6	5,543,124	17	Phenylethanal_ UR ^*^	1,77E-05	14	1

*The most significant association detected for each compound is reported. CH, chromosome; hap., haplotypic; UR, unroasted beans; R, roasted beans*.

* *Biochemical compounds known for floral notes, bp, base pair*.

The map showing the results for compounds of the L-phenylalanine degradation pathway (Supplementary Figure 6) shows several interesting results.

One hundred and eleven co-locations between different VOCs were also observed on all chromosomes. An example of co-localisation was observed between 4-hydroxyacetophenone (UR) and acetophenone (UR) on chromosome 5 (Figure 4B).

Thirteen co-locations between at least one aroma VOC and one sensory trait were observed on chromosomes 1, 2, 8, and 9 (Supplementary Figure 6).

### Significant Associations Were Identified for the Biochemical Compounds Involved in Other Pathways

Several areas of association were highlighted for seven other compounds known also to have a floral taste: ethyl dodecanoate (R), guaiacol (UR and R), hexyl acetate (UR), furfural (UR and R), propyl acetate (R), and nonanal (UR). One hundred and seventeen association zones were detected for guaiacol (UR) against zero for guaiacol (R). Twelve association zones were observed for furfural (UR) compared to 30 for furfural (R) (Table 5). The variation in hexyl acetate concentration is very high compared to other compounds. On the other hand, the genetic explanation for the variation in the concentration of propyl acetate is very weak compared to the other characteristics of this study (4%).

**Table 5:** Most significant associations for biochemical compounds related to other pathways.

**CH**	**Position of the association peak (bp)**	**N° hap bloc**	**Traits**	***p-*Value of the strongest association**	**Explanation rate of the trait of the strongest association (%)**	**Associations detected**
3	31,273,182	57	Ethyl dodecanoate _ R ^*^	6,60E-11	36	50
10	5,308,832	26	Furfural_ R ^*^	3,15E-07	22	13
7	4,776,442	NA	Furfural_ UR ^*^	3,70E-08	24	5
4	737,310	NA	Guaiacol_ UR ^*^	1,60E-09	28	54
7	10,459,413	31	Hexyl acetate_ UR ^*^	1,01E-138	67	94
9	37,557,289	54	Nonanal_ UR ^*^	2,45E-07	22	9
9	3,653,985	14	Propyl acetate _ R ^*^	2,42E-14	4	41

*The most significant association detected for each compound is reported. CH, chromosome; hap, haplotypic; UR, unroasted beans; R, roasted beans*.

* *Biochemical compounds known for floral notes, bp, base pair*.

### Candidate Genes Potentially Involved in the Formation of the Floral Aroma

Of the 393 association zones exposed, 27 with candidate genes with predicted functions were identified.

#### Candidate Genes Linked to the Terpene Biosynthesis Pathway

Candidate genes related to the terpene biosynthetic pathway were found on chromosomes 1, 2, 5, 7, 9, and 10. The association zone number and candidate genes are reported in Supplementary Figure 7; Supplementary Table 3; and Table 6.

**Table 6:** Candidate genes identified for terpene biosynthesis pathway.

**N° asso**	**CH**	**Position of candidate gene (bp)**	**Position of the pic of association (bp)**	**Candidate Gene function**	**Trait in association**
1	1	1,883,959	2,368,915	*Geranylgeranyl pyrophosphate synthase, chloroplastic*	Epoxylinalool-R
2	1	3,173,783	3,379,361	*Cytochrome P450 81E8*	Epoxylinalool (R), floral note lightwood
2	1	3,179,883	3,379,361	*Cytochrome P450 81E8*	Epoxylinalool (R), floral note lightwood
3	1	6,026,043	6,130,253	*Cytochrome P450 78A7*	Epoxilinalool (R)
4	2	7,549,475	7,324,500	*Dehydrodolichyl diphosphate synthase 6*	Cis ocimene co-eluted with ethyl hexanoate (UR), floral perfume
4	2	7,551,259	7,324,500	*Dehydrodolichyl diphosphate synthase 6*	Cis ocimene co-eluted with ethyl hexanoate (UR), floral perfume
4	2	7,553,795	7,324,500	*Dehydrodolichyl diphosphate synthase 6*	Cis ocimene co-eluted with ethyl hexanoate (UR), floral perfume
4	2	7,572,073	7,324,500	*Putative Dehydrodolichyl diphosphate synthase 6*	Cis ocimene co-eluted with ethyl hexanoate (UR), floral perfume
5	2	8,257,841	8,389,914	*Probable 3-hydroxyisobutyryl-CoA hydrolase 2*	Linalool cis pyranic oxide (UR)
6	5	32,749,861	33,303,465	*Cytochrome P450 89A2*	Linalool (UR) and linalool trans furanic oxide (UR)
6	5	33,057,632	33,303,465	*Cytochrome P450 89A9*	Linalool (UR) and linalool trans furanic oxide (UR)
6	5	33,064,477	33,303,465	*Cytochrome P450 89A2*	Linalool (UR) and linalool trans furanic oxide (UR)
6	5	33,073,996	33,303,465	*Cytochrome P450 89A2*	Linalool (UR) and linalool trans furanic oxide (UR)
6	5	33,094,200	33,303,465	*Cytochrome P450 89A2*	Linalool (UR) and linalool trans furanic oxide (UR)
6	5	33,099,009	33,303,465	*Cytochrome P450 89A2*	Linalool (UR) and linalool trans furanic oxide (UR)
7	7	6,346,577	6,181,185	*Putative Probable terpene synthase 9*	Linalool cis pyranic oxide (UR)
7	7	6,365,007	6,181,185	*Probable terpene synthase 9*	Linalool cis pyranic oxide (UR)
7	7	6,380,614	6,181,185	*Putative Probable terpene synthase 9*	Linalool cis pyranic oxide (UR)
8	9	791,968	749,365	*3-hydroxyisobutyryl-CoA hydrolase-like protein 2, mitochondrial*	Epoxylinalool (R)
9	10	6,317,543	6,167,221	*Probable terpene synthase 9*	Linalool cis pyranic oxide (UR)

*asso., associations; CH, chromosome; R, roasted beans; UR, unroasted beans*.

On chromosome 1, three association zones contain candidate genes. Association zone 1 (805,132–2,445,782 bp) linked to epoxylinalool (R) contains a gene coding for a “*Geranylgeranyl pyrophosphate synthase, chloroplastic*.” This enzyme allows the synthesis of geranylgeranyl pyrophosphate in chloroplasts. This compound is a precursor of terpenes. As the monoterpene biosynthesis pathway is located in the plastids, the indication of chloroplastic synthesis seems to confirm the correspondence to another compound derived from linalool also synthesised in Chloroplast (Ying and Qingping, 2006; Feng et al., 2014). Association zone 2 (3,083,032–3,398,183 bp) linked to epoxylinalool (R) and the floral note lightwood contains two candidate genes encoding a “*Cytochrome P450 81E8*.” Cytochrome P450 has been identified to be responsible for the synthesis of epoxylinalool from linalool in kiwifruit (Chen et al., 2010). Association zone 3 (5,940,526–6,204,028 bp) linked to epoxylinalool (R) contains a candidate gene encoding a “*Cytochrome P450 78A7*.”

On chromosome 2 (Supplementary Figure 7), two association zones contain candidate genes. Association zone 4 (7,324,500–7,617,242 bp) linked to cis ocimene co-eluted with ethyl hexanoate (UR) and floral perfume contains four genes encoding a “*Dehydrodolichyl diphosphate synthase 6*” (DDS 6) in Figure 4A. Dehydrodolichyl diphosphate synthase 6 allows the synthesis of dehydrodolichyl diphosphate, one of the precursors of which is geranyl diphosphate, the main precursor of the monoterpene biosynthesis pathway. The synthesis of dehydrodolichyl diphosphate could thus compete with the synthesis of cis-ocimene and explain the association with this compound as well as with the floral perfume, which is a taste attributed to several monoterpenes (linalool, epoxylinalool, ocimene). Association zone 5 (8,239,972–8,416,672 bp) linked to linalool cis pyranic oxide (UR) contains a gene encoding a “*Probable 3-hydroxyisobutyryl-CoA hydrolase 2*.” The enzyme 3-hydroxyisobutyryl-CoA hydrolase 2 can enable the production of acetyl-CoA by releasing a CoA. Acetyl-CoA is a precursor of the mevalonate biosynthetic pathway that allows the production of geranyl diphosphate (Kreck et al., 2003; Miziorko, 2011).

**Figure 7:**
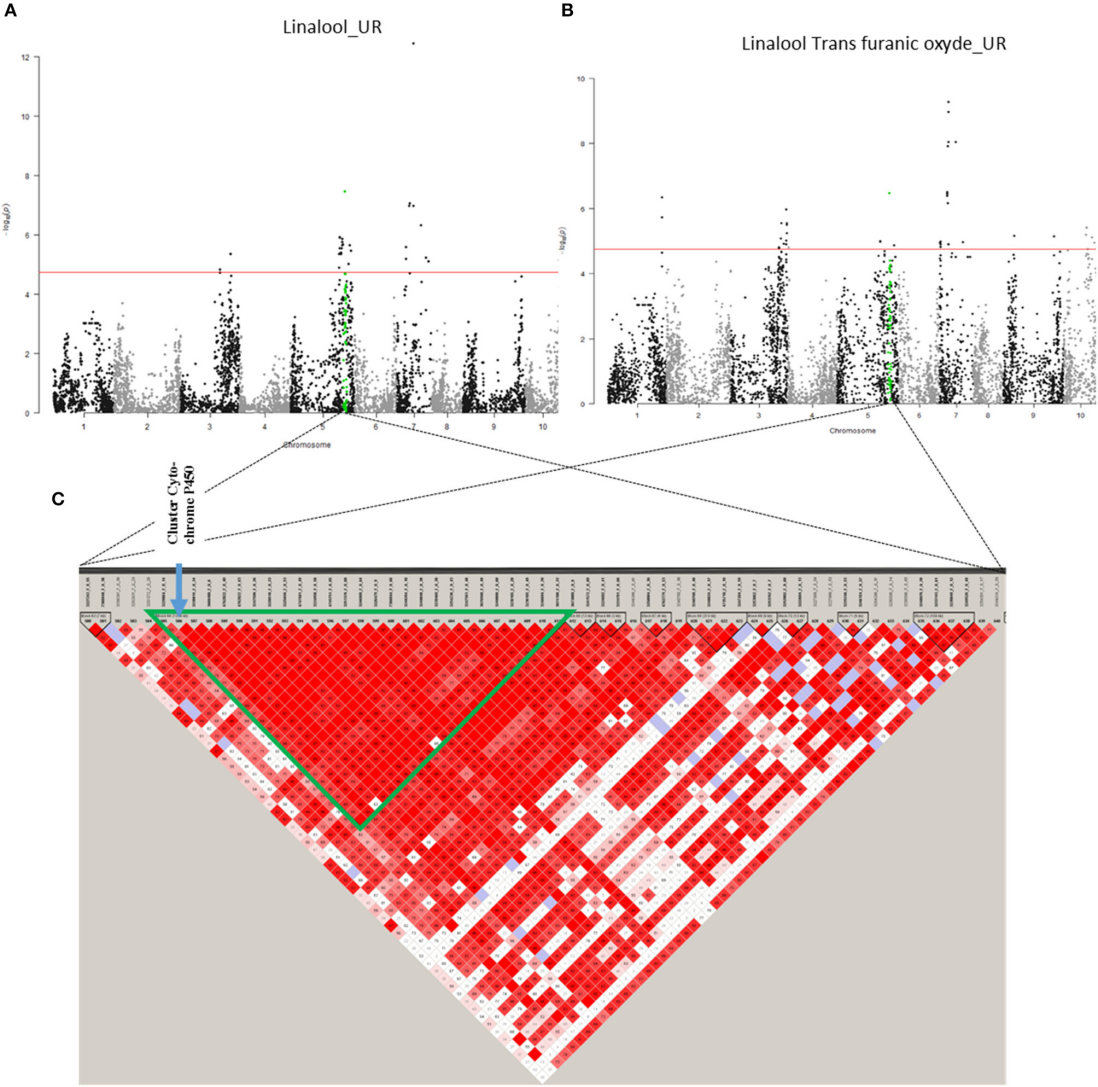
Co-localisation between linalool (UR) and trans furanic oxide (UR) with candidate genes. **(A)** Manhattan plot representing association results for the Linalool (UR) trait, revealed by GLM-MAF5 method. **(B)** Manhattan plot representing association results for linalool trans furanic oxide (UR) trait, revealed by GLM-MAF5 method. **(C)** Heat map of a part of chromosome 5. The common region of association is represented by a green triangle.

On chromosome 5, only association region 6 (32,660,102–33,718,239 bp) contains candidate genes. It is linked to linalool (UR) and linalool trans furanic oxide (UR) and contains six candidate genes, five of which are known to code for “*Cytochrome P450 89A2*” and one for “*Cytochrome P450 89A9*” (Figure 7; Supplementary Figure 7). The presence of cytochrome P450 could explain the associations with linalool and trans furanic oxide linalool as they would allow the transformation of linalool into epoxylinalool (Chen et al., 2010).

On chromosome 7 (Supplementary Figure 7), only association zone 7 (6,128,106–6,410,151 bp) contains candidate genes. It is linked to linalool cis pyranic oxide (UR) and contains three genes encoding “*Probable terpene synthase 9*.” Terpene synthases 9 are known to be involved in the synthesise of linalool, one of the precursors of linalool cis pyranic oxide (Cseke et al., 1998).

On chromosome 9 (Supplementary Figure 7), only association zone 8 (713,588–857,818 bp) contains a candidate gene. It is linked to epoxylinalool (R) and contains a gene encoding a “*3-hydroxyisobutyryl-CoA hydrolase-like protein 2, mitochondrial*.” This enzyme is involved in the mevalonate biosynthetic pathway, one of the biosynthetic pathways leading to the formation of geranyl diphosphate, a key compound in the monoterpene biosynthetic pathway (Lamarti et al., 1994).

On chromosome 10 (Supplementary Figure 7), the association zone 9 (6,023,982–6,718,126 bp) linked to linalool cis pyranic oxide (UR) contains a gene coding for “*Probable terpene synthase 9*.” This enzyme is known to synthesise linalool, which could enable the synthesis of linalool cis pyranic oxide.

#### Candidate Genes Linked to the L-Phenylalanine Degradation Pathway

In a second step, candidate genes linked to the L-phenylalanine degradation pathway were found on chromosomes 1, 2, 4, 5, 7, 8, 9, and 10. The association zone number and candidate genes are reported in Supplementary Figure 6; Supplementary Table 3; and Table 7.

**Table 7:** Candidate genes identified for L-phenylalanine degradation pathway.

**N° asso**	**CH**	**Position of candidate gene (bp)**	**Position of the pic of association (bp)**	**Gene function**	**Trait in association**
10	1	1,102,658	2,430,002	*Aldehyde dehydrogenase family 3 member F1*	1-Phenylethyl acetate (R), benzaldehyde (R), and cinnamaldehyde (R)
11	1	3,107,999	3,379,361	*Probable cinnamyl alcohol dehydrogenase 7/8*	1-Phenylethyl acetate (R), phenylethyl acetate co-eluted with 2-ethylphenol (R), acetophenone (R), benzaldehyde (R), cinnamaldehyde (R), and the floral note lightwood
11	1	3,112,047	3,379,361	*Probable cinnamyl alcohol dehydrogenase*	1-Phenylethyl acetate (R), phenylethyl acetate co-eluted with 2-ethylphenol (R), acetophenone (R), benzaldehyde (R), cinnamaldehyde (R), and the floral note lightwood
12	1	6,039,217	5,940,526	*Shikimate kinase 1, chloroplastic*	1-Phenylethyl acetate (R) and cinnamaldehyde (R)
13	1	7,124,110	6,855,567	*Alcohol dehydrogenase 1*	1-Phenylethyl acetate (R), phenylethyl acetate co-eluted with 2-ethylphenol (R), acetophenone (R), benzaldehyde (R), and cinnamaldehyde (R)
13	1	7,131,266	6,855,567	*Alcohol dehydrogenase 1*	1-Phenylethyl acetate (R), phenylethyl acetate co-eluted with 2-ethylphenol (R), acetophenone (R), benzaldehyde (R), and cinnamaldehyde (R)
14	2	7,436,835	7,453,377	*Aminotransferase ALD1*	Acetophenone (NT and R), benzaldehyde (R), benzyl alcohol (UR), cinnamaldehyde (R), and the floral perfume note
15	4	22,566,348	22,503,297	*Acetyltransferase NSI*	1-Phenylethyl acetate and cinnamaldehyde (R)
16	4	26,741,963	26,876,494	*3-ketoacyl-CoA thiolase 2, peroxisomal*	1-Phenylethyl acetate (R)
16	4	26,715,852	26,876,494	*Chalcone synthase 2*	1-Phenylethyl acetate (R)
17	4	27,604,955	27,507,597	*2-hydroxyisoflavanone dehydratase*	Floral perfume
17	4	27,608,704	27,507,597	*2-hydroxyisoflavanone dehydratase*	Floral perfume
18	4	28,270,492	28,285,175	*Probable aldo-keto reductase 1*	1-Phenylethyl acetate (R)
19	5	1,328,453	1,353,636	*Aldehyde oxidase GLOX*	4-Hydroxy acetophenone (UR), acetophenone (UR), and benzyl acetate (UR)
20	5	1,431,497	1,380,802	*Aldo-keto reductase family 4 member C9*	4-Hydroxy acetophenone (UR), acetophenone (UR), benzyl acetate (UR), and cinnamaldehyde (R)
20	5	1,435,043	1,380,802	*Aldo-keto reductase family 4 member C9*	4-Hydroxy acetophenone (UR), acetophenone (UR), benzyl acetate (UR), and cinnamaldehyde (R)
20	5	1,438,324	1,380,802	*Aldo-keto reductase family 4 member C9*	4-Hydroxy acetophenone (UR), acetophenone (UR), benzyl acetate (UR), and cinnamaldehyde (R)
20	5	1,441,067	1,380,802	*Aldo-keto reductase family 4 member C9*	4-Hydroxy acetophenone (UR), acetophenone (UR), benzyl acetate (UR), and cinnamaldehyde (R)
20	5	1,444,916	1,380,802	*Aldo-keto reductase family 4 member C8*	4-Hydroxy acetophenone (UR), acetophenone (UR), benzyl acetate (UR), and cinnamaldehyde (R)
20	5	1,450,213	1,380,802	*Aldo-keto reductase family 4 member C8*	4-Hydroxy acetophenone (UR), acetophenone (UR), benzyl acetate (UR), and cinnamaldehyde (R)
21	5	2,978,188	2,732,709	*Phenylalanine ammonia-lyase*	Cinnamaldehyde (R)
22	5	30,446,215	30,471,918	*Alcohol dehydrogenase-like 6*	Benzaldehyde (R)
23	7	2,055,963	2,092,063	*GDSL esterase/lipase At1g28570*	4-Hydroxy acetophenone (UR), acetophenone (UR), and benzyl acetate (UR)
24	8	1,199,801	1,148,435	*3-ketoacyl-CoA synthase 4*	Floral note wood resin
25	8	2,170,173	2,251,806	*GDSL esterase/lipase EXL3*	Cinnamaldehyde (R)
26	8	6,559,785	6,751,843	*Acetyltransferase At1g77540*	Acetophenone (UR) and benzyl acetate (UR)
26	8	6,570,129	6,751,843	*Caffeic acid 3-O-methyltransferase*	Acetophenone (UR) and benzyl acetate (UR)
26	8	6,581,002	6,751,843	*Caffeic acid 3-O-methyltransferase*	Acetophenone (UR) and benzyl acetate (UR)
27	8	15,370,133	14,498,544	*Putative O-acyltransferase WSD1*	Benzaldehyde (R), benzyl acetate (UR), cinnamaldehyde (R), and orange blossom note
27	8	15,402,378	14,498,544	*Putative O-acyltransferase WSD1*	Benzaldehyde (R), benzyl acetate (UR), cinnamaldehyde (R), and orange blossom note
28	8	18,679,641	19,249,315	*Putative GDSL esterase/lipase At1g29670*	Benzyl acetate (UR)
29	9	5,605,457	6,010,658	*GDSL esterase/lipase EXL3, putative*	Benzyl alcohol (UR) and the floral note green vegetative
29	9	6,069,175	6,010,658	*3-hydroxyisobutyryl-CoA hydrolase-like protein 3, mitochondrial*	Benzyl alcohol (UR) and the floral note green vegetative
30	9	23,325,443	23,302,911	*Feruloyl CoA ortho-hydroxylase 2*	Acetophenone (R), benzaldehyde (R), and benzyl acetate (UR)
31	10	5,303,984	5,308,832	*Putative 4-coumarate–CoA ligase-like 5*	1-Phenylethyl acetate (R), benzyl acetate (R), phenylethyl acetate co-eluted with 2-ethylphenol (R), to acetophenone (R), benzaldehyde (R,) and cinnamaldehyde (R)
6	5	32,749,861	33,303,465	*Cytochrome P450 89A2*	2-Phenylethanol (UR)
6	5	33,057,632	33,303,465	*Cytochrome P450 89A9*	2-Phenylethanol (UR)
6	5	33,064,477	33,303,465	*Cytochrome P450 89A2*	2-Phenylethanol (UR)
6	5	33,073,996	33,303,465	*Cytochrome P450 89A2*	2-Phenylethanol (UR)
6	5	33,094,200	33,303,465	*Cytochrome P450 89A2*	2-Phenylethanol (UR)
6	5	33,099,009	33,303,465	*Cytochrome P450 89A2*	2-Phenylethanol (UR)

*asso., Associations; CH, chromosome; R, Roasted beans; UR, unroasted beans*.

On chromosome 1, four association zones contain candidate genes. Association zone 10 (805,132–2,445,782 bp) linked to 1-phenylethyl acetate (R), benzaldehyde (R), and cinnamaldehyde (R) contains a gene coding for an “*Aldehyde dehydrogenase family 3 member F1*.” This enzyme could be responsible for the transformation of benzaldehyde into benzoic acid. The presence of this enzyme could compete with the production of cinnamaldehyde or 1-phenylethyl acetate (Figure 6; Lapadatescu et al., 2000). Association zone 11 (3,083,032–3,398,183 bp) linked to 1-phenylethyl acetate (R), phenylethyl acetate co-eluted with 2-ethylphenol (R), acetophenone (R), benzaldehyde (R), cinnamaldehyde (R), and the floral note lightwood, contains two candidate genes encoding a “*Probable cinnamyl alcohol dehydrogenase*.” These enzymes are known to transform cinnamaldehyde into cinnamyl alcohol (Wyrambik and Grisebach, 1975). According to another study, “*Probable cinnamyl alcohol dehydrogenase*” has the ability to remove hydrogen from cinnamyl alcohol to convert it to cinnamaldehyde. Cinnamyl alcohol is known to have a floral, cinnamon, and balsamic taste (Steinhaus et al., 2009), which may be associated with the floral note lightwood. The association zone 12 (5,940,526–6,204,028 bp) linked to 1-phenylethyl acetate (R) and cinnamaldehyde (R) contains a gene encoding a “*Shikimate kinase 1, chloroplastic*.” The shikimate biosynthesis pathway allows the synthesis of phenylalanine, a precursor of 1-phenylethyl acetate and cinnamaldehyde (Tohge et al., 2013). The association zone 13 (6,834,165–7,942,921), linked to 1-phenylethyl acetate (R), phenylethyl acetate co-eluted with 2-ethylphenol (R), acetophenone (R), benzaldehyde (R), and cinnamaldehyde (R), contains two genes coding for an “*Alcohol dehydrogenase 1*.” Alcohol dehydrogenase is necessary for the degradation of benzaldehyde to benzyl alcohol or vice versa, which are both compounds with a fruity taste. The other compounds in association in this area are upstream of this degradation reaction, which could explain their associations (Lapadatescu et al., 2000).

On chromosome 2 (Supplementary Figure 6), only association region 14 (7,324,500–7,617,242 bp) contains candidate genes. It is linked to acetophenone (NT and R), benzaldehyde (R), benzyl alcohol (UR), cinnamaldehyde (R), and the floral perfume note and contains a candidate gene coding for an “*ALD1 Aminotransferase*.” Several aminotransferases have been identified in the shikimate biosynthesis pathway that allows the synthesis of L-phenylalanine (Tohge et al., 2013).

On chromosome 4 (Supplementary Figure 6), four association zones contain candidate genes. Association region 15 (22,435,678–22,617,119 bp) linked to 1-phenylethyl acetate and cinnamaldehyde (R) contains a gene encoding an “*NSI acetyltransferase*.” The acetyl transferase NSI has the function of acetylating histones. It is likely to play a role in regulating the expression of genes for the synthesis of 1-phenylethyl acetate or cinnamaldehyde. Association zone 16 (26,703,951–27,146,370 bp) linked to 1-phenylethyl acetate (R) contains two candidate genes coding for: a “*Chalcone synthase 2*” and a “*3-ketoacyl-CoA thiolase 2, peroxisomal*.” Chalcone synthases participate in the flavonoid and isoflavonoid biosynthesis pathway that follows the degradation of phenylalanine to CA (Pyrzynska and Biesaga, 2009). A ketoacyl-Coa thiolase is required for the synthesis of benzoyl-CoA (Amano et al., 2018), which can be the basis for phenylbenzoate synthesis. The association zone 17 (27,507,597–27,608,727 bp) linked to the floral perfume contains two genes encoding a “*2-hydroxyisoflavanone dehydratase*.” 2-hydroxyisoflavanone is part of the isoflavonoid biosynthesis pathway. Its transformation could compete with the synthesis of compounds known to have a floral taste such as acetophenone or 2-phenylethanol (Pyrzynska and Biesaga, 2009). The association zone 18 (28,257,730–28,352,788 bp) linked to 1-phenylethyl acetate (R) contains a gene coding for a “*Probable aldo-keto reductase 1*.” An acetaldehyde reductase may be required for the synthesis of 1-phenylethanol from acetophenone, the probable precursor of 1-phenylethyl acetate (Dong et al., 2012).

**Figure 8:**
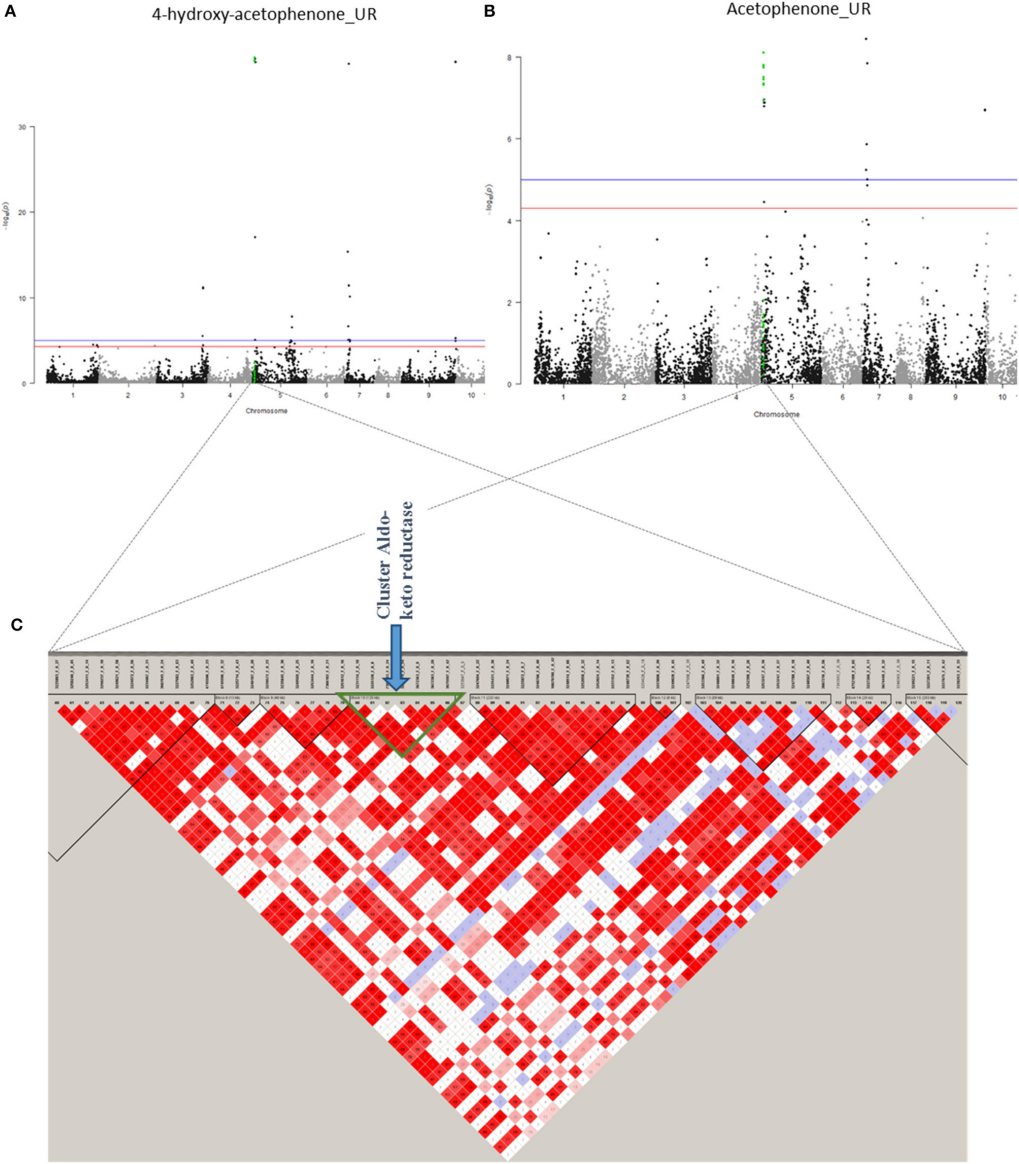
Co-localisation between 4-hydroxy-acetophenone (UR) and acetophenone (UR) with candidate genes. **(A)** Manhattan plot representing association results for the trait 4-hydroxy-acetophenone (UR). **(B)** Manhattan plot representing association results for acetophenone (UR). **(C)** Heat map of a part of chromosome 5. The common region of association is represented by a green triangle.

On chromosome 5, five association zones contain candidate genes. Association region 19 (1,326,444–1,374,494 bp) linked to 4-hydroxy acetophenone (UR), acetophenone (UR), and benzyl acetate (UR) contains a candidate gene encoding a “*GLOX Aldehyde oxidase*.” An aldehyde oxidase is in some cases responsible for the oxidation of phenylacetaldehyde to phenylacetate, both of which are part of the L-phenylalanine degradation pathway (Kücükgöze and Leimkühler, 2018). Association zone 20 (1,380,802–1,510,054 bp) linked to 4-hydroxy acetophenone (UR), acetophenone (UR), benzyl acetate (UR), and cinnamaldehyde (R) contains six candidate genes, four of which code for an *Aldo-keto reductase family 4 member C9* and two for an *Aldo-keto reductase family 4 member C8* (Figure 8; Supplementary Figure 6). An acetaldehyde reductase may be required for the synthesis of 1-phenylethanol from acetophenone, a probable precursor of 1-phenylethyl acetate (Dong et al., 2012). The association zone 21 (2,674,400–3,039,540 bp) linked to cinnamaldehyde (R) contains a gene coding for a *Phenylalanine ammonia-lyase*. This enzyme is known to transform L-phenylalanine into CA, which is the precursor of cinnamaldehyde (Lapadatescu et al., 2000). The association zone 22 (30,407,214–30,473,075 bp) linked to benzaldehyde (R) contains a gene coding for an *Alcohol dehydrogenase-like 6*. This enzyme could degrade benzaldehyde to benzyl alcohol. Association zone 6 (32,660,102–33,718,239 bp) is linked to 2-phenylethanol (UR) (the same to terpene association zone 6). It contains six genes, five of which code for *Cytochrome P450 89A2* and one for *Cytochrome P450 89A9*. Cytochrome P450 has redox activities. Several of these reactions are involved in the synthesis of 2-phenylethanol (Lapadatescu et al., 2000).

On chromosome 7 (Supplementary Figure 6), only association zone 23 (1,894,664–2,092,063 bp) contains a candidate gene. It is linked to 4-hydroxy acetophenone (UR), acetophenone (UR), and benzyl acetate (UR) and contains a gene encoding a *GDSL esterase/lipase At1g28570*. A lipase/esterase may be required for the formation of benzyl acetate from benzyl alcohol or the synthesis of 1-phenyl acetate from 1-phenyl ethanol (Mäki-Arvela et al., 2008; Melo et al., 2017).

On chromosome 8 (Supplementary Figure 6), five association zones contain candidate genes. Association zone 24 (1,121,979–1,520,555 bp) linked to the floral note wood resin contains a candidate gene encoding a *3-ketoacyl-CoA synthase 4*. This enzyme is involved in the transformation of a very long chain of acyl-CoA into acetyl-CoA which can itself be transformed into ketones (Tong et al., 2006). Since this zone of associations is linked to the floral note wood resin, this gene can perhaps lead to the synthesis of ketones known to have a floral taste like acetophenone. Association zone 25 (2,021,946–2,268,116 bp) linked to cinnamaldehyde (R) contains a candidate gene encoding a *GDSL esterase/lipase EXL3*. An esterase/lipase may be required as previously discussed for the formation of benzyl acetate from benzyl alcohol or the synthesis of 1-phenylehtyl acetate (Mäki-Arvela et al., 2008; Melo et al., 2017). The synthesis of these compounds could compete with the synthesis of cinnamaldehyde. The association zone 26 (6,533,242–6,978,549 bp) linked to acetophenone (UR) and benzyl acetate (UR) is linked to three genes, two of which code for *Caffeic acid 3-O-methyltransferase* and one for *Acetyltransferase At1g77540*. Caffeic acid 3-O-methyltransferase has the role of transforming caffeic acid into ferulic acid and can thus compete with the synthesis of acetophenone or benzyl acetate (Tu et al., 2010). An acetyltransferase is required to convert benzyl alcohol to benzyl acetate (Hao et al., 2014). This function may explain the associations with acetophenone, which requires a common benzyl alcohol precursor for synthesis. Association zone 27 (14,444,953–15,439,624 bp) linked to benzaldehyde (R), benzyl acetate (UR), cinnamaldehyde (R), and orange blossom note contains two genes encoding a *Putative O-acyltransferase WSD1*. This enzyme allows the synthesis of a “wax ester” from long-chain fatty alcohol. It could allow the synthesis of a “wax ester” with a floral taste of orange blossom type or contribute to this aromatic note. The association zone 28 (17,816,898–19,249,315 bp) linked to benzyl acetate (UR) contains a candidate gene coding for a *Putative GDSL esterase/lipase At1g29670* that may play a role in the degradation of benzyl acetate (Mäki-Arvela et al., 2008; Melo et al., 2017).

On chromosome 9 (Supplementary Figure 6), two association zones contain candidate genes. Association zone 29 (5,327,028–6,165,415 bp) linked to benzyl alcohol (UR) and the floral note green vegetative contains two genes: one coding for *3-hydroxyisobutyryl-CoA hydrolase-like protein 3, mitochondrial* and one for *GDSL esterase/lipase EXL3, putative*. The 3-hydroxyisobutyryl-CoA hydrolase-like enzyme could lead to the synthesis of terpenes with floral tastes as described above. It could thus explain the association with the floral green vegetative taste. Lipase may be required for the formation of benzyl acetate from benzyl alcohol (Melo et al., 2017). The enzyme encoded by the GDSL esterase/lipase gene EXL3, putative could compete with the synthesis of benzyl alcohol. Association zone 30 (23,101,222–23,892,356 bp) linked to acetophenone (R), benzaldehyde (R), and benzyl acetate (UR) contains a gene encoding a *Feruloyl CoA ortho-hydroxylase 2*. Ferulic acid has CA as a precursor, as do acetophenone, benzaldehyde, and benzyl acetate. The activity of this enzyme could therefore compete with the synthesis of these compounds.

On chromosome 10 (Supplementary Figure 6), one association zone contains candidate genes. Association zone 31 (5,153,882–5,419,006 bp) linked to 1-phenylethyl acetate (R), benzyl acetate (R), phenylethyl acetate co-eluted with 2-ethylphenol (R), to acetophenone (R), benzaldehyde (R), and cinnamaldehyde (R) contains a candidate gene encoding a *Putative 4-coumarate–CoA ligase-like 5*. The activity of this enzyme could compete with the synthesis of compounds associated with this region as it could induce a transformation of CA to coumaric acid.

## Discussion

This study contributes to highlighting the importance of cocoa genetic background in the aroma composition of cacao products. The GWAS analyses revealed a large number of associations. Several are related to VOCs known for their floral aromas, others are related to compounds, without floral aroma, but involved in the biosynthesis of these aromatic compounds, and others are related to the perception of sensory notes.

### Determination of Associations Area

The confidence interval of the association zones was determined using haplotypic blocks. This method gives an idea of the size of the association zone as a function of the linkage disequilibrium of the population, which seems biologically logical. However, in some cases, this limit may underestimate the true size of the association, as it is certainly the case on chromosome 1 for the epoxylinalool (R) trait (Supplementary Figure 7) where we see hot spots of associations extending over the first seven megabases. In cases where there is a cluster of very close association zones, it is legitimate to ask whether the method of determining the association zones is not too stringent.

### Insights into the Genetic Architecture of Floral Aromas in Cocoa

Genome-Wide Association Study analysis, two main biosynthesis pathways of compounds known for their floral notes seem to be involved in cocoa floral aromas: the monoterpene synthesis pathway and the L-phenylalanine degradation pathway. These biosynthesis pathways have already been identified in other such as grapes or its derivative wine as important contributors to their floral aromas (Ferreira et al., 1997; Mateo and Jiménez, 2000). Some of the association zones contain candidate genes directly involved in the synthesis of the associated compound, or candidate genes involved upstream in the biosynthetic pathway. The presence of these genes increases the probability that the detected association is not a false positive. The GWAS analyses revealed several genes that appear to be involved in the synthesis of compounds known to have a floral taste and could thus be involved in the variation of floral tastes. Candidate genes coding for enzymes are the most obvious, but other types of genes may be involved in cocoa floral taste such as certain transcriptional factors that could activate or repress several biosynthetic pathways at the same time.

Some associations linked to compounds from the same biosynthesis pathway have been co-localised. Roasting has been suggested to play a role in the transformation of these compounds (Jinap et al., 1998). This could explain some of the co-localisation observed in this study, for example, in the terpene biosynthesis pathway the degradation of linalool to epoxylinalool or vice versa (co-localisation on chromosome 5), the transformation of cis pyranic oxide linalool to epoxylinalool or the opposite (co-localisation on chromosomes 4 and 10). Roasting may also play a role in the transformation of compounds in the L-phenylalanine degradation pathway as, for example: 4-hydroxy acetophenone to acetophenone or vice versa (co-localisation on chromosomes 7 and 10), the transformation of benzyl acetate into benzaldehyde or the opposite (co-locations on chromosomes 2, 5, 7, 8, 9, and 10), and the transformation of benzyl alcohol into benzaldehyde or vice versa (co-locations on chromosomes 2, 3, 4, 5, 6, 8, and 10).

Other associations give information on a balance between the presence of aromatic and non-aromatic compounds of the same biosynthetic pathway: suggesting that an enzyme could be responsible for the transformation of one of these compounds into another and thus influence the flavour as observed in roses by Farhi et al. (2010). The presence of certain odours would thus depend on the activation or repression of the enzyme responsible for the synthesis of the compound with the floral aroma. This is the case, for example, for an area on chromosome 1 associated with cinnamaldehyde and the floral note lightwood containing a gene coding for a “Probable cinnamyl alcohol dehydrogenase.” When this enzyme is active, it would allow the transformation of cinnamaldehyde into cinnamyl alcohol. There would then be a possible accumulation of cinnamyl alcohol known to have a floral note. When this enzyme is not active, cinnamaldehyde, which has a spicy (cinnamon) taste, would accumulate. Other areas of association suggest that a similar system has been put in place: this is the case for the co-locations between 1-phenylethyl acetate and acetophenone on chromosomes 1, 6, 9, and 10 where a gene coding for an esterase/lipase has been detected in nearby location for association zones in chromosome 1, 6, and 9 (Supplementary Table 3). If that gene would be active, an accumulation of 1-phenylethyl acetate known to have a fruity odour would be possible. Otherwise, a possible accumulation of acetophenone, also known to have a floral note would be obtained. This is also the case for the co-localisation between benzyl acetate and benzyl alcohol on chromosome 2. A cluster of genes coding for an esterase/lipase and a gene with an acetyltransferase function was detected close to co-location (Supplementary Table 3). In this case, if the enzyme is active, an accumulation of benzyl alcohol known to have a sweet taste could be observed. If the enzyme is inactive, a possible accumulation of benzyl acetate known to have a jasmine note could be observed. In the case of co-locations between 4-hydroxy acetophenone and acetophenone on chromosomes 5, 7, and 9 the enzyme transforming 4-hydroxy acetophenone into acetophenone has not been characterised. The candidate gene must have a hydroxylase function that allows the addition of the hydroxyl function on carbon number 4. Two genes (*2-nonaprenyl-3-methyl-6-methoxy-1, 4-benzoquinol hydroxylase*, and *Abscisic acid 8'-hydroxylase 2*) with this function been identified close to the association zones on chromosomes 7 and 9 (Supplementary Table 3).

The position of the most significant association zones for the same compound may be different if this compound has been detected in roasted or unroasted beans. This is the case for benzyl acetate, acetophenone, benzaldehyde, furfural, and linalool (Tables 3–5). This difference can be explained by the response to two different phenomena: during fermentation, the enzymes responsible for the synthesis of compounds would be activated. A “classical” synthesis would then be carried out in the bean. Whereas, during roasting, the thickness of the shell or the size of the bean could play a role in the chemical conditions of the bean such as temperature or pH and thus influence the degradation of certain aromatic compounds. In that case, the detection of association would depend also on the location of genes involved in the bean structure and size. It is also possible that the difference is due to the presence of precursors that allow the genesis of aromatic compounds during roasting.

This is not the case for all compounds. On the contrary, 2-phenylethanol dosed in roasted and unroasted beans has peaks of very close associations and there are also co-locations between acetophenone related associations dosed in roasted and unroasted beans on chromosomes 2, 6, and 9 confirming the importance of these areas in the genesis of these compounds.

The formation of an aroma as well as its perception depends on a large number of conditions. An aromatic note is generally composed of a combination of several VOCs at different concentrations (Pérez-Silva et al., 2006). Aromatic traits, therefore, have a high probability of being polygenic, which is consistent with the large number of associations that have been found in this study. The expression of an aromatic note also depends on the matrix in which VOCs are contained (Afoakwa et al., 2008). The production of these compounds by plants also depends on their environment (Baldwin, 2010). These factors therefore partly explain why large number of associations was found.

The synthesis of a flavour is therefore due to many external parameters but also the genetic background of the *T. cacao* trees (Luna et al., 2002; Afoakwa et al., 2008). Due to its multigenic determinism, the total variance of a compound is the result of many small associations, each of which would explain, a small part of the genetic variance. Once these small associations are combined, they could explain a large part of the genetic variance. In this case, some associations may contain only one associated marker, as is the case for linalool on chromosome 2. It is also possible that some associations do not cross the significance threshold and are therefore not identified. This hypothesis suggests that some associations with certain VOCs have not been revealed, explaining why the analysis of some compounds known to have a floral taste does not reveal an association zone as for guaiacol (R).

### Role of Fermentative Micro-Organisms in Cocoa Flavour Synthesis

The analysis of three other compounds known to have a floral taste belonging to the family of esters did not detect zones of associations: ethyl 2-hydroxyhexanoate (R), ethylphenyl acetate (UR), and ethyl hexanoate (UR). These compounds present after fermentation and before roasting could also be synthesised by yeasts during fermentation (Soles et al., 1982). In this case, no area of association can be found as this would depend on the micro-organisms population and not on the cocoa seeds. The non-detection of association zones can also be due partially to the pollination of the mother tree made by a mix of progenitors. While genotyping is done on the mother tree, phenotyping (VOC assay and sensory analysis) is done on the beans, hybrids between the mother tree and male pollinators, which could lead to a partial discrepancy between genetic and phenotypic data. Currently, it is not possible to genotype and phenotype individually each bean.

Volatile organic compounds (VOCs) produced by plants are involved in various processes and often released for defence, signalling, or pollinator attraction purposes (Baldwin, 2010). Volatile organic compounds belong to different biochemical families such as terpenes. They are notably involved in direct and indirect defence against insects (Martin et al., 2002) and micro-organisms (Pichersky et al., 1995). Compounds of the terpene family are recognised as a molecular signal in many interactions between plants and various other species, particularly in competition reactions, in the presence of herbivores or pathogenic microorganisms, but also the presence of beneficial insects (Langenheim, 1994; Bohlmann et al., 1998). The same is true for certain phenolic compounds such as acetophenone or 4-hydroxyacetophenone that could be involved in defence mechanisms (Parent et al., 2018), which has also been observed for furfural (Palmqvist et al., 1999; Miller et al., 2009).

During fermentation, the change in environment and chemical composition of the medium induced by yeasts and bacteria can be taken as a threat and cause the seed to react. Then, they could release VOCs to defend themselves and would be responsible for the synthesis of VOCs involved in fine flavour, as suggested by Sabau et al. (2006) who observed an increase in the expression of the gene coding for linalool synthase during fermentation. Also, a strong increase in the concentration of linalool, epoxylinalool, and 2-phenylethanol has also been observed during fermentation in aromatic fine cocoa beans by other authors (Cevallos-Cevallos et al., 2018).

If cocoa beans use VOCs as a defence mechanism against external microorganisms such as fermentative yeasts, lactic bacteria, or acetic bacteria, some questions remain unanswered: by which mechanisms do they detect such microorganisms? Knowing that different types of yeast have been identified according to the place of fermentation (Schwan and Wheals, 2004), we can also ask ourselves whether certain types of yeast or microorganisms are more favourable to this activation. Another hypothesis is that the presence of microorganisms and the transformations they induce (change in pH, synthesis of unknown compounds in the seed, etc.) induce the synthesis of VOCs. In this case, VOCs could be triggered in the absence of microorganisms.

## Conclusions and Perspectives

The perception of an aroma and the sensorial analyses is a difficult task. They, therefore, depends on a large number of conditions, including the perception threshold of aromatic molecules. The presence of a molecule is therefore not synonymous with the perception of its taste. Similarly, regions of the genome identified as being associated with the content of biochemical compounds do not mean that these compounds are involved in the flavour of cocoa. Additional analyses are necessary to validate the involvement of these molecules in the formation of taste such as gas chromatography coupled to olfactometry (GCO) analyses for example. Knowing the main molecules responsible for the floral taste as well as the mechanisms of synthesis and degradation of the compounds during fermentation and roasting could also, in the long term, allow the adaptation of the roasting process (temperatures and roasting time) to preserve the most fragile aromatic compounds. Knowledge of the biosynthesis pathway of cocoa aromatic compounds could provide a better mastering of the parameters of fermentations allowing the synthesis of these molecules.

The identification of these molecules and their biosynthetic pathway within the cocoa tree is complex. A genomic selection approach could allow early prediction of aroma traits for the search of cocoa trees having good aroma potential, especially as certain genetic variation could explain a large extend of biochemical compounds in the beans. In this case, a marker-assisted selection could be envisaged in the selected programmes to make it easier for the selection of the cocoa trees aromatic quality.

## Data Availability Statement

The datasets presented in this study can be found in online repositories. The names of the repository/repositories and accession number(s) can be found in the article/Supplementary Material.

## Ethics Statement

The studies involving human participants did not require approval in line with regional/national guidelines. The patients/participants provided their written informed consent to participate in this study.

## Author Contributions

EC, CL, and RL conceived the experiment. J-CJ and AS conducted biochemical analyses. ES carried out sensorial analyses. OF carried out DNA experiments. KC, J-CJ, AS, RB, CL, FD, SA, and XA analysed data. KC, RB, and CL wrote the manuscript. All authors contributed to the article and approved the submitted version.

## Funding

This study was funded by the United States Department of State (U.S. Foreign Ministry), the U.S. Embassy, Quito, and the U.S. Department of Agriculture (USDA-ARS) with the agreement n° 58-4001-2-F128 and the MUSE Amazcacao project with the reference ANR-16-IDEX-0006.

## Conflict of Interest

The authors declare that the research was conducted in the absence of any commercial or financial relationships that could be construed as a potential conflict of interest.

## Publisher's Note

All claims expressed in this article are solely those of the authors and do not necessarily represent those of their affiliated organizations, or those of the publisher, the editors and the reviewers. Any product that may be evaluated in this article, or claim that may be made by its manufacturer, is not guaranteed or endorsed by the publisher.
