# Supplementary figures and images for: Two Main Biosynthesis Pathways Involved in the Synthesis of the Floral Aroma of the Nacional Cocoa Variety
Source: Front Plant Sci. 2021 Sep 24;12:681979. doi: 10.3389/fpls.2021.681979 (PMC8498224; doi:10.3389/fpls.2021.681979)

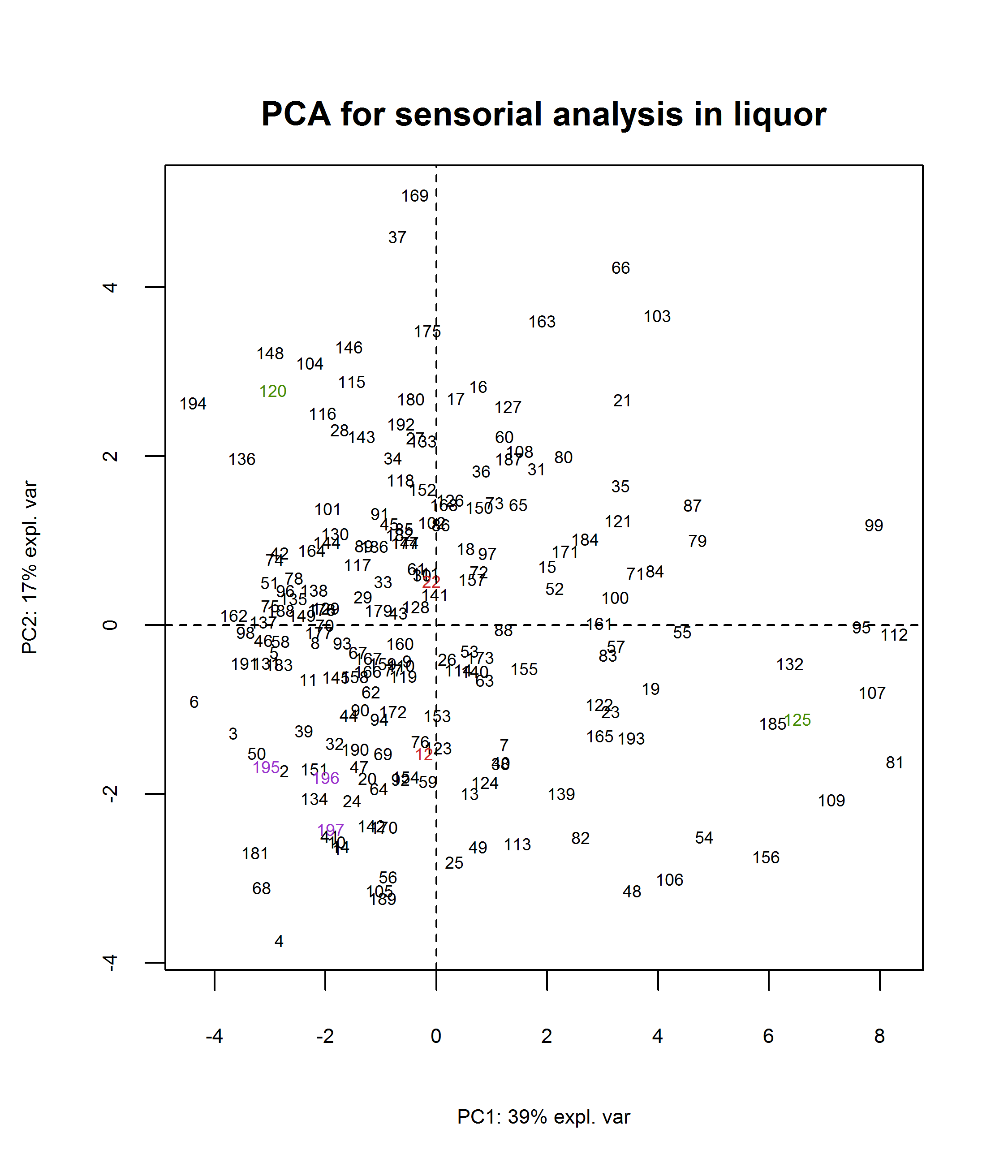

Supplement: Supplementary file 4 [file Image_1.TIFF]

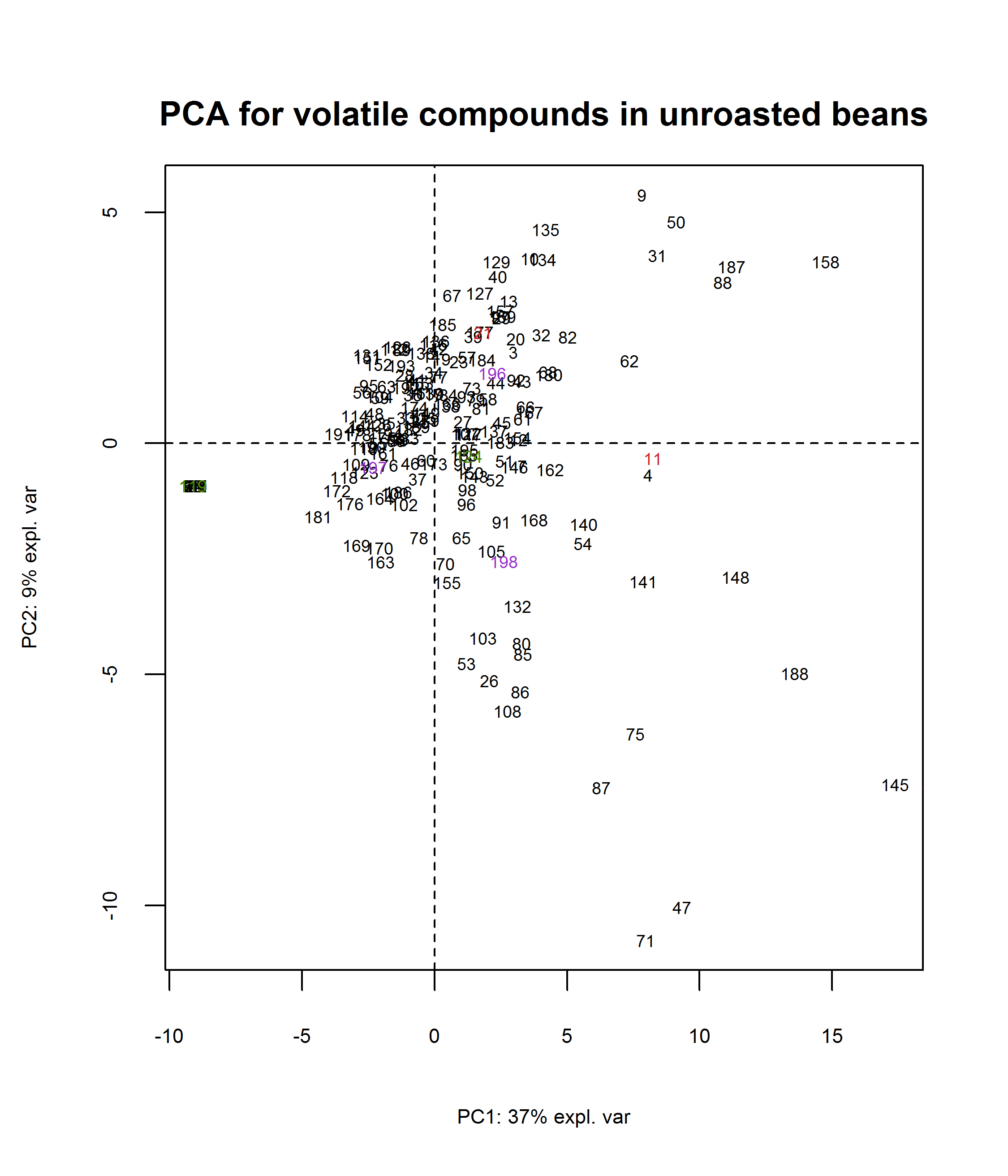

Supplement: Supplementary file 5 [file Image_2.TIFF]

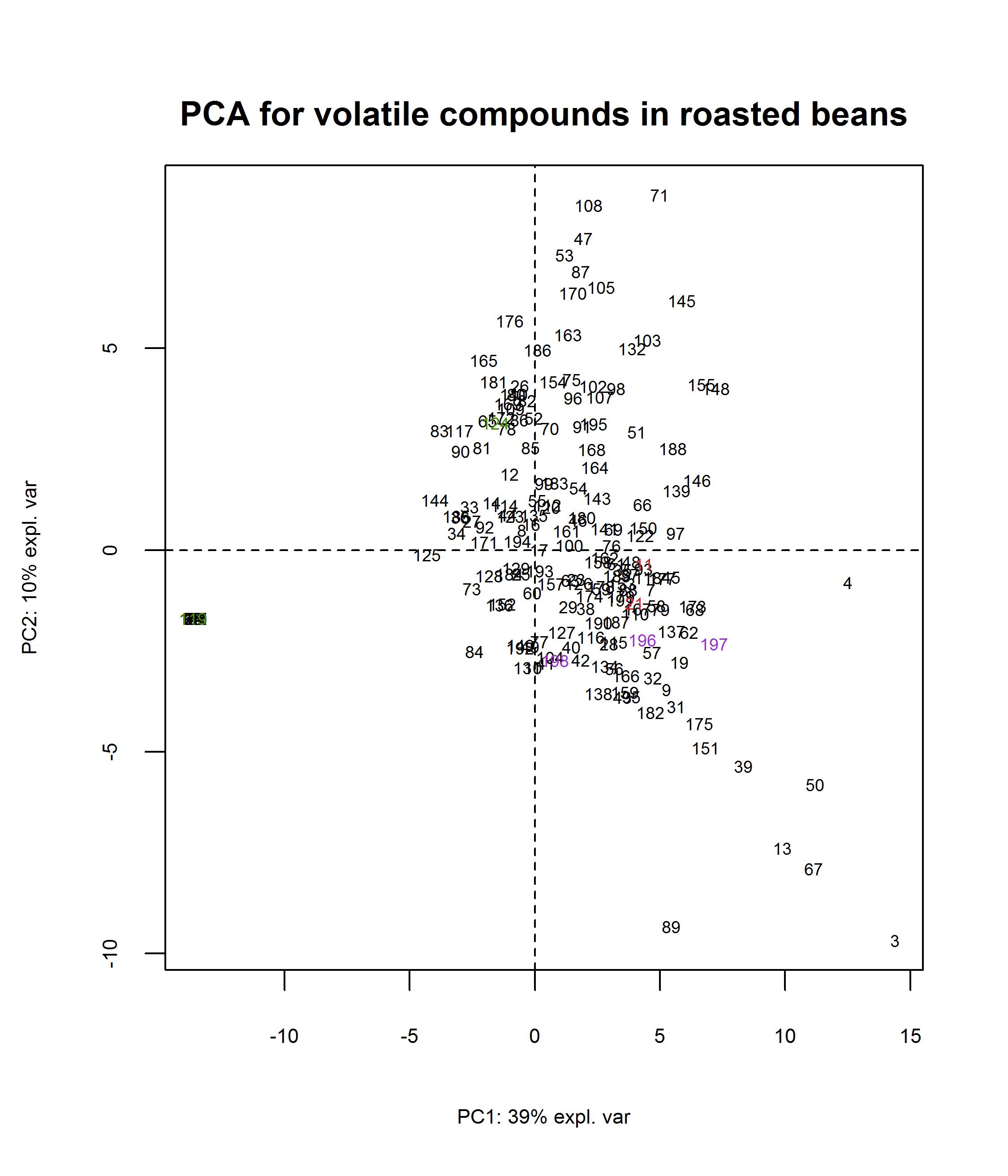

Supplement: Supplementary file 6 [file Image_3.TIFF]

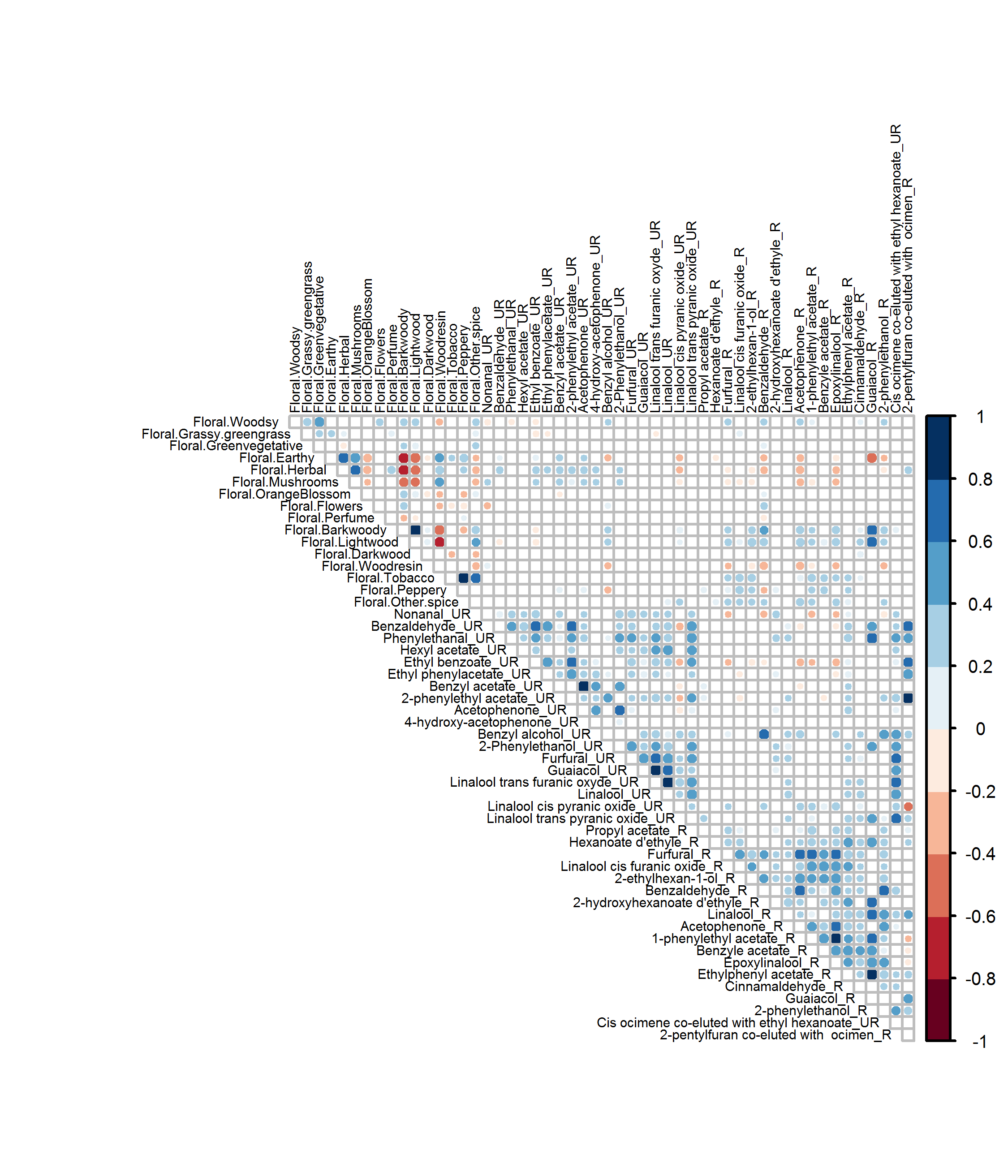

Supplement: Supplementary file 7 [file Image_4.TIFF]

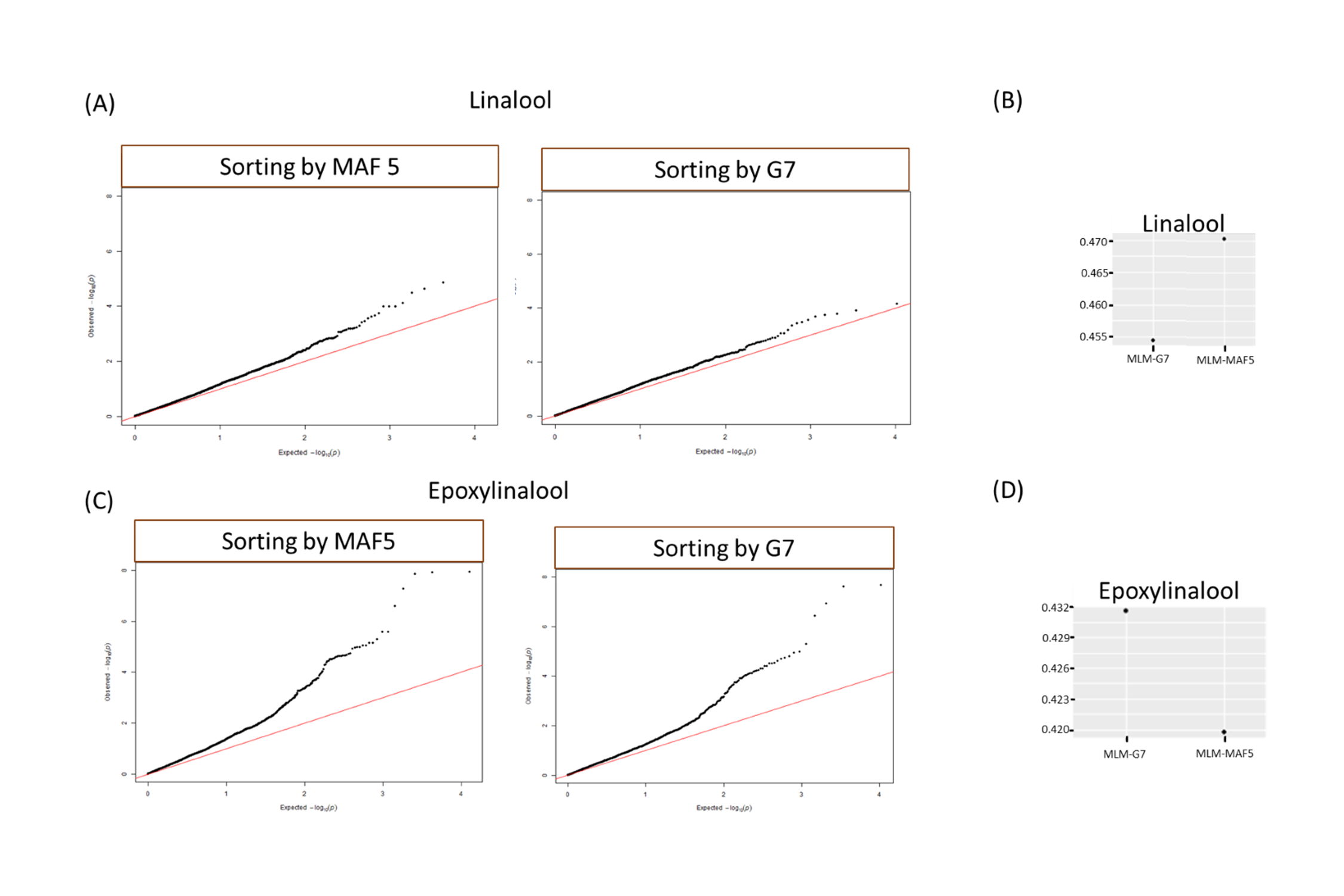

Supplement: Supplementary file 8 [file Image_5.TIF]

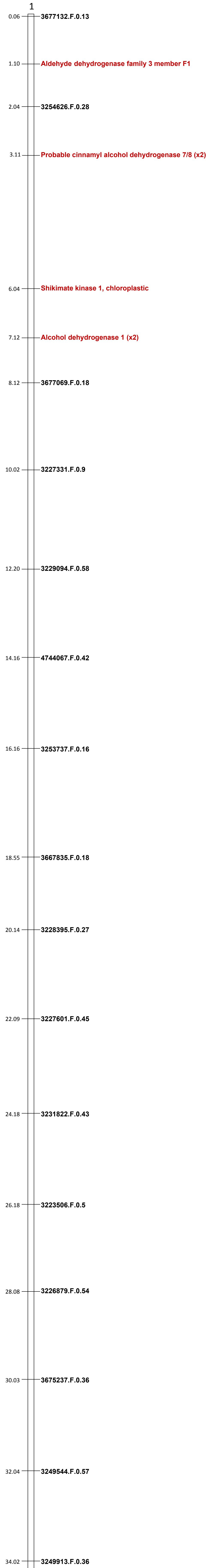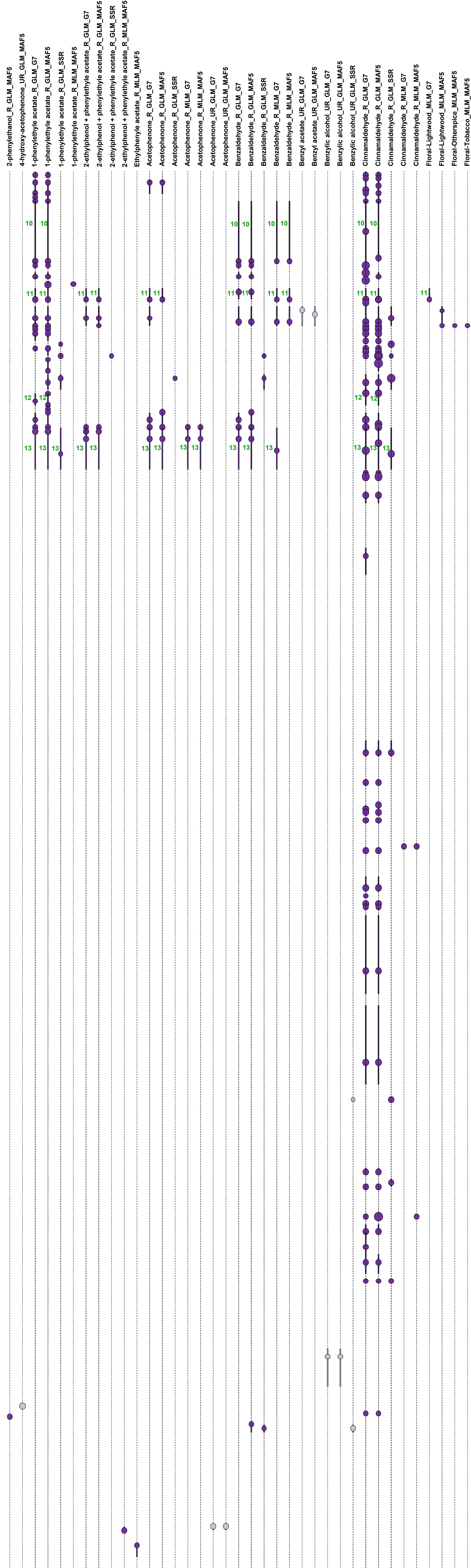

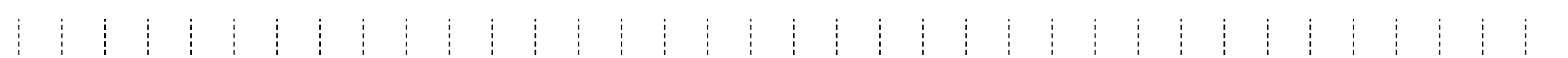

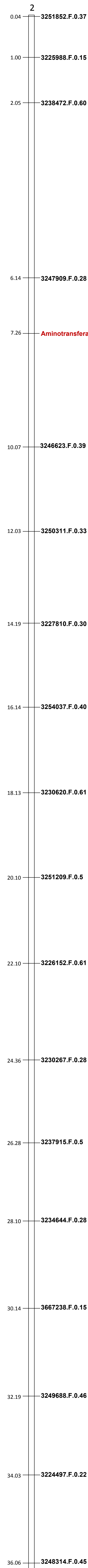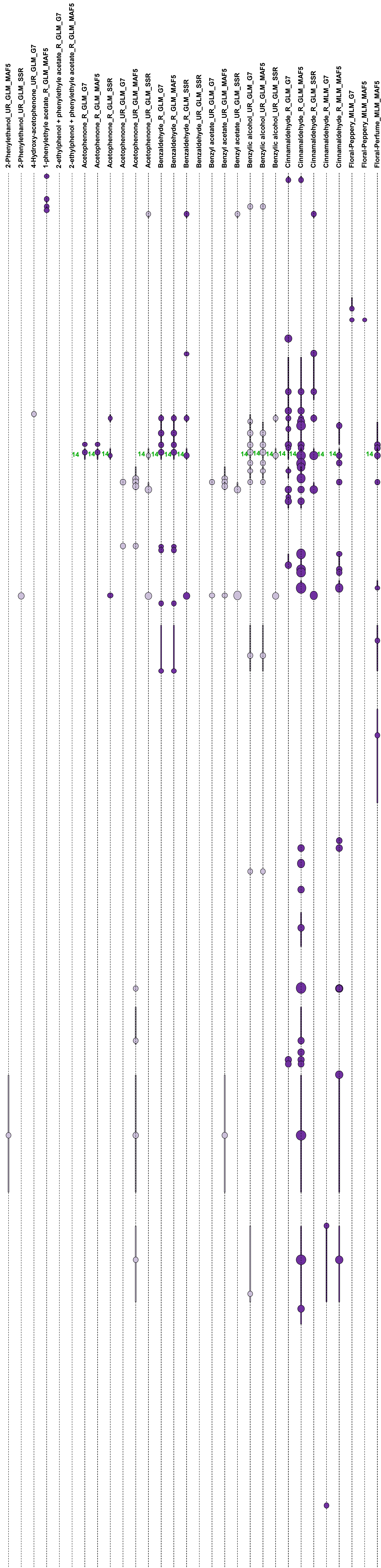

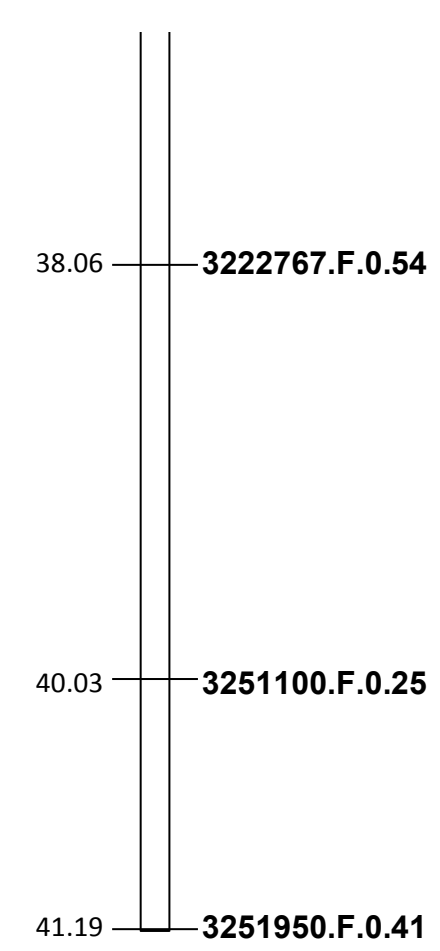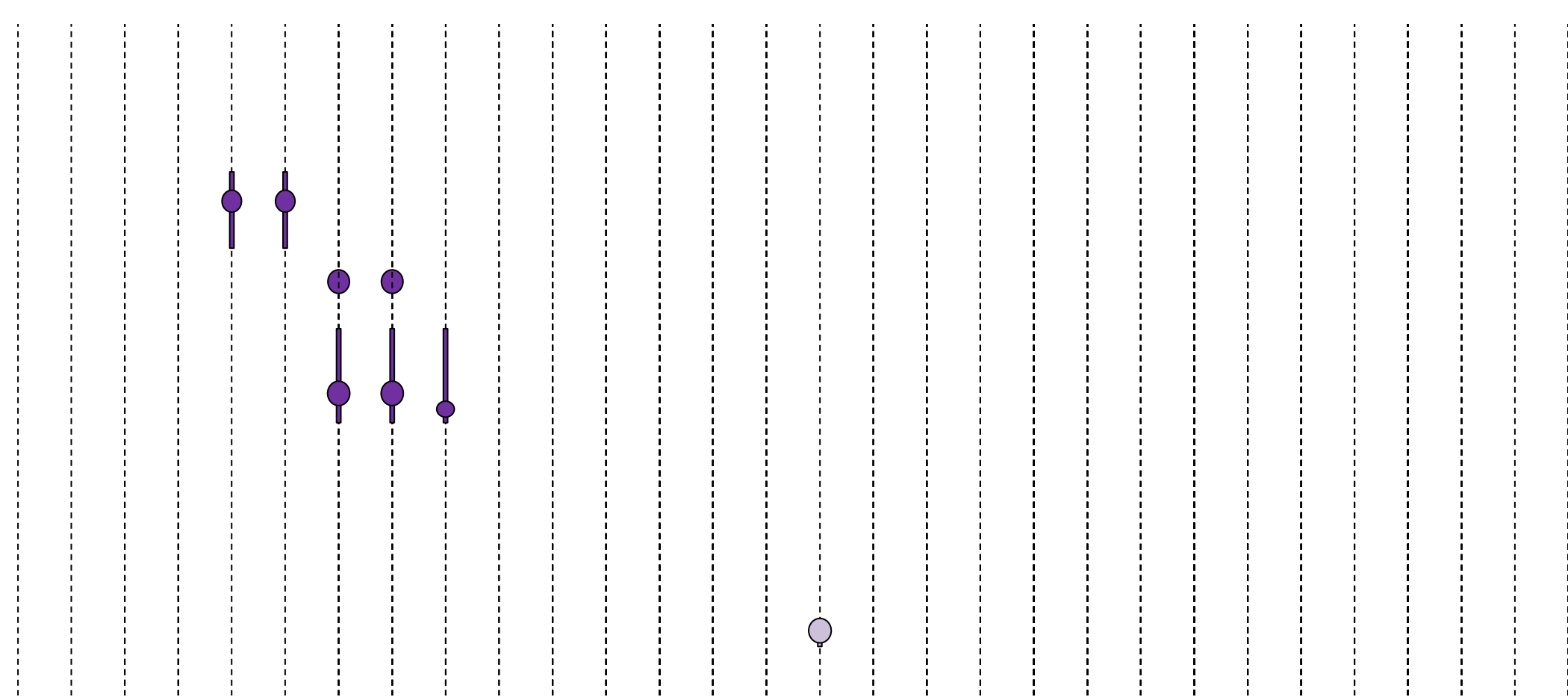

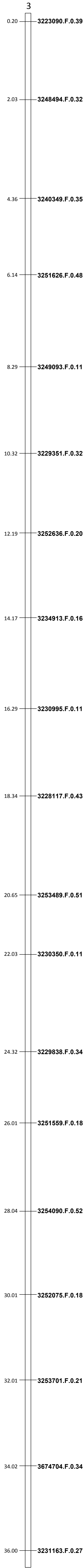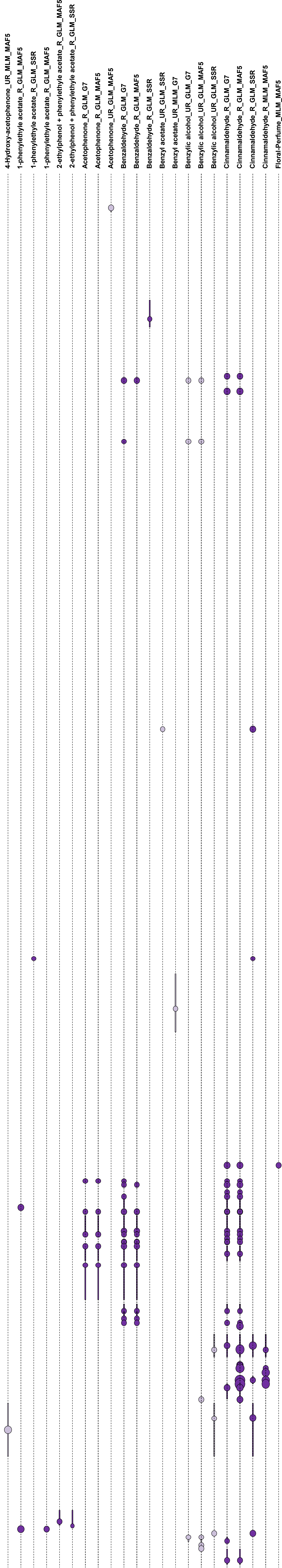

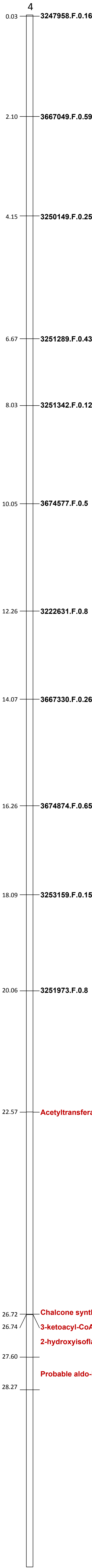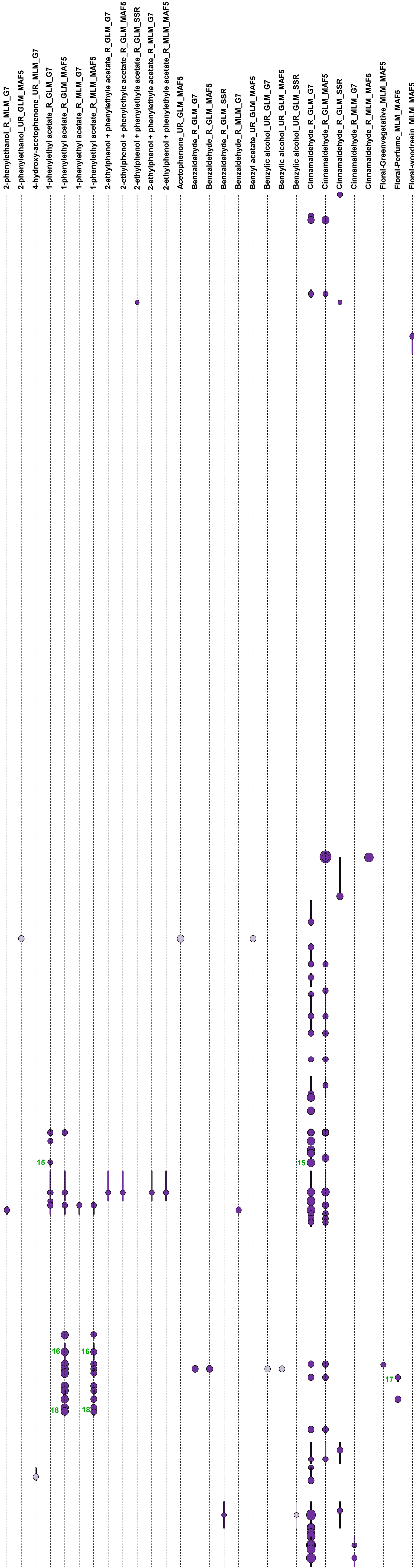

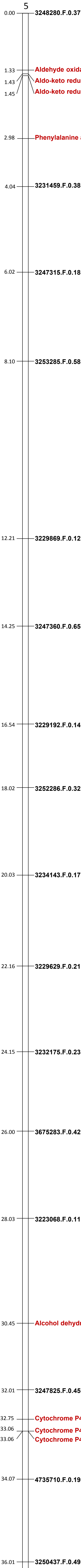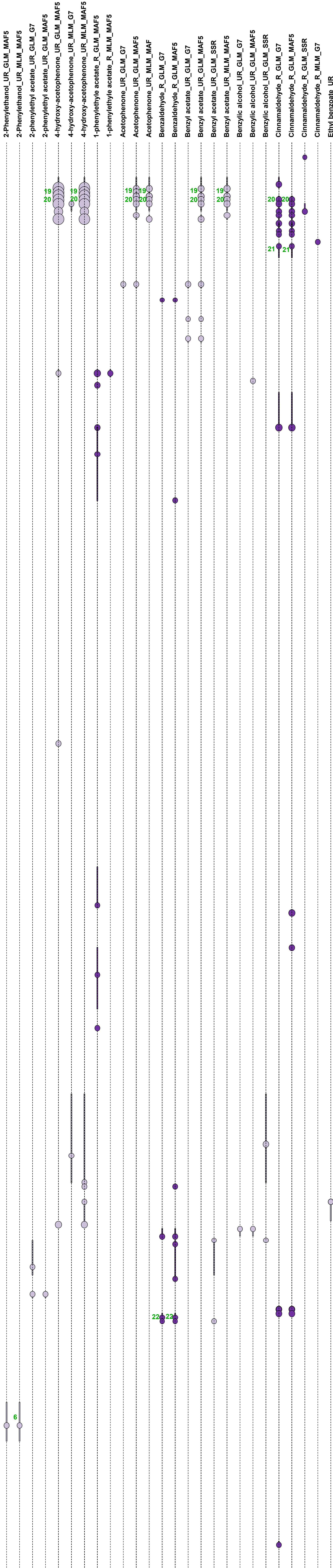

38.01 — **3250743.F.0.9**

39.35 — **18681789.F.0.46**

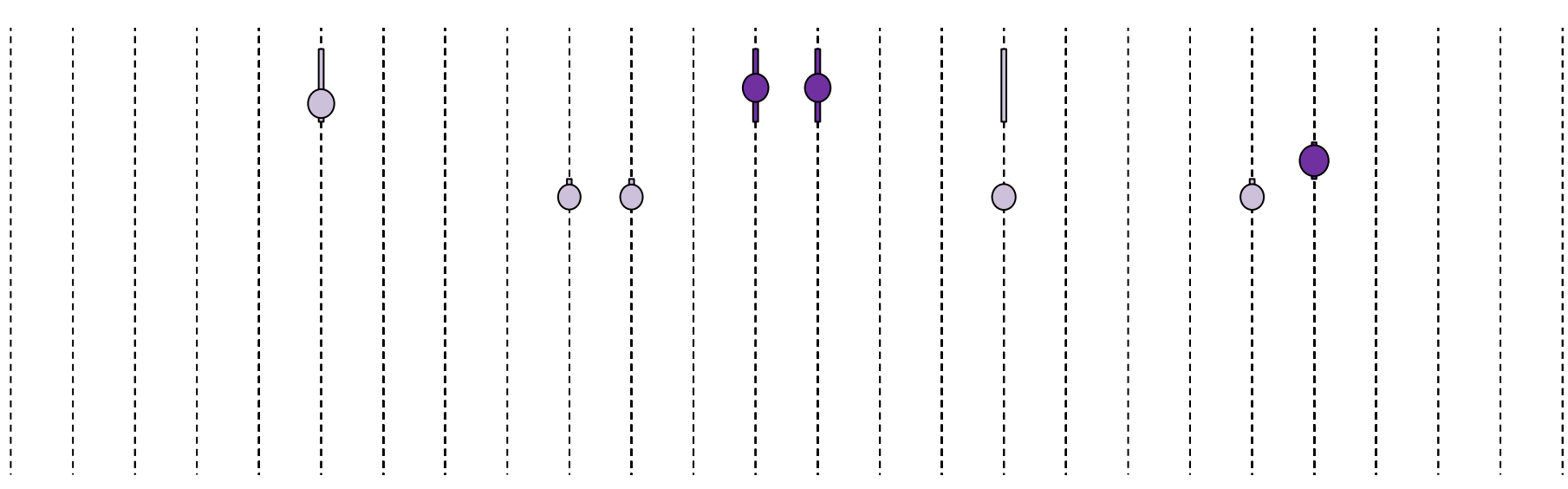

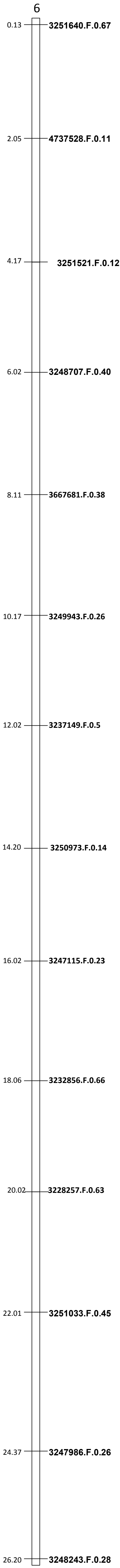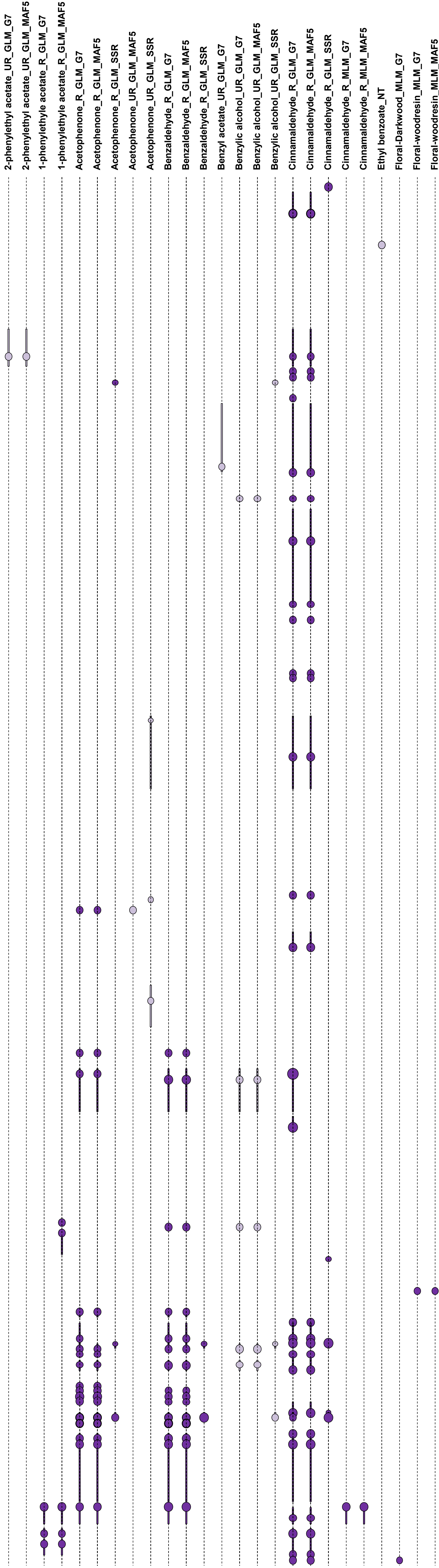

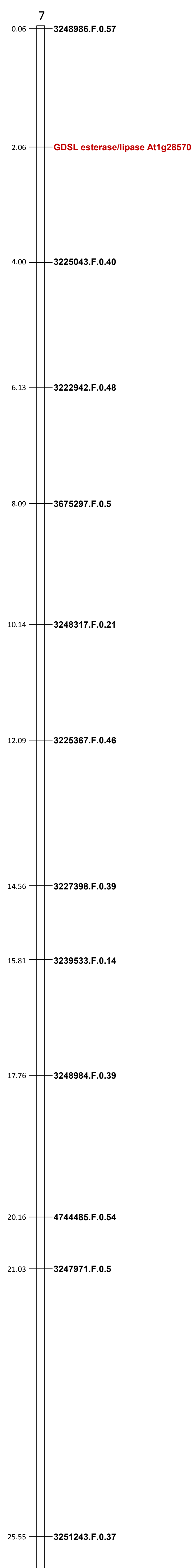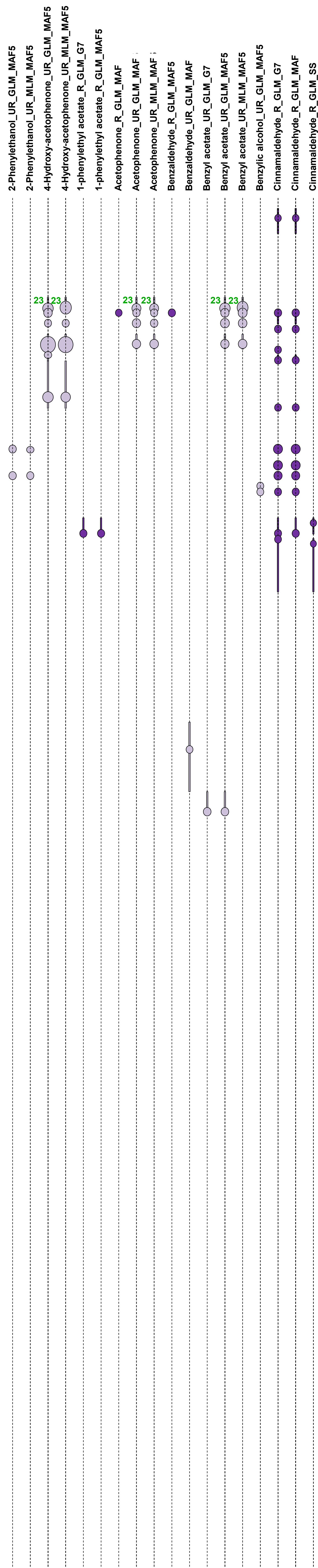

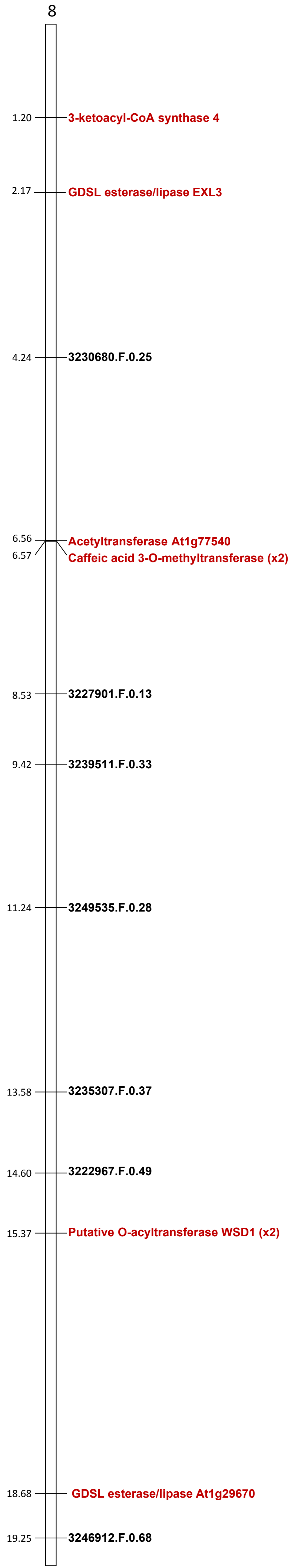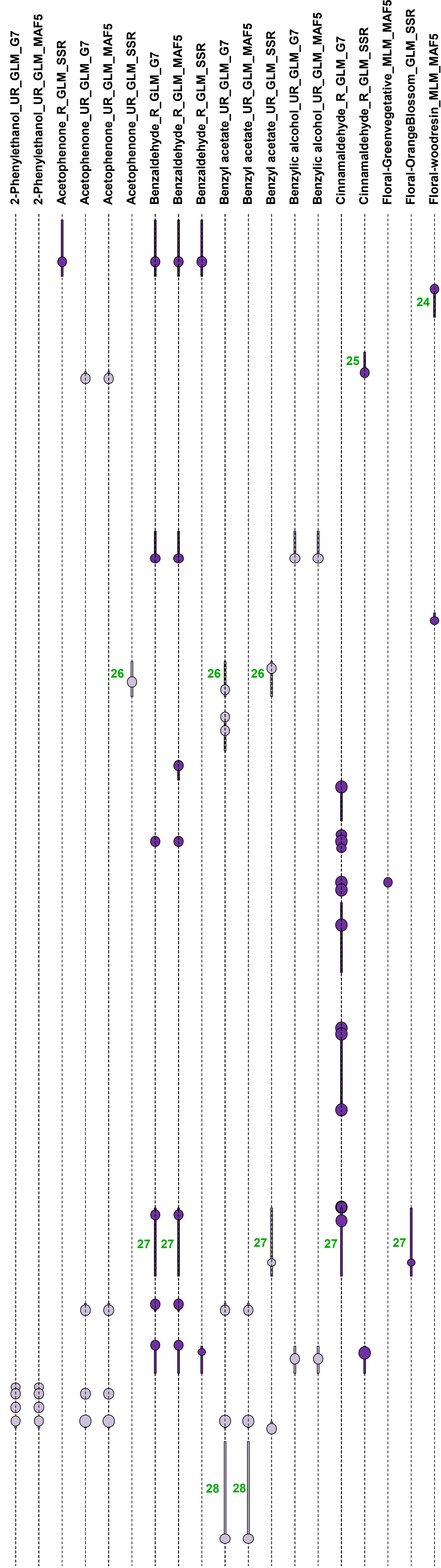

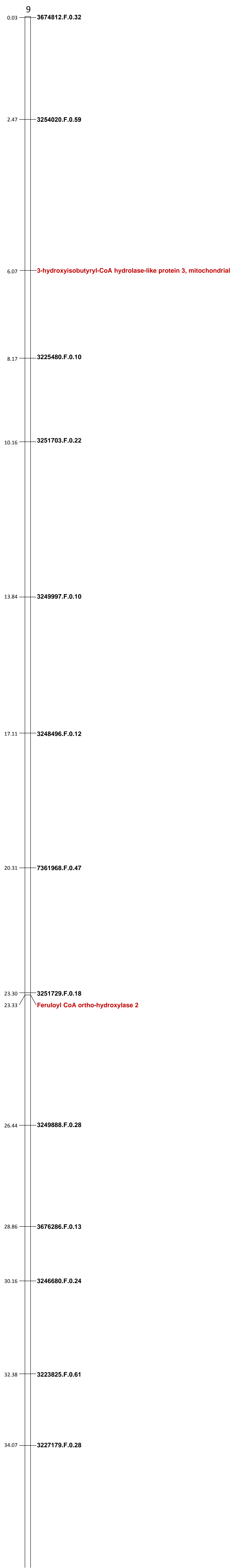

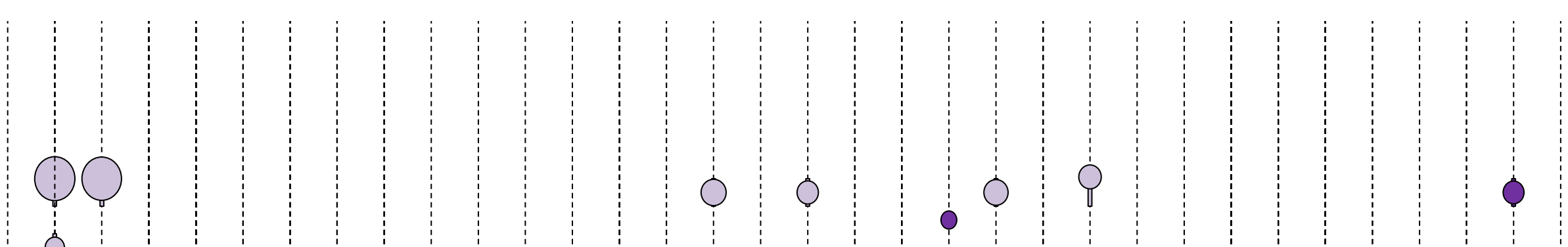

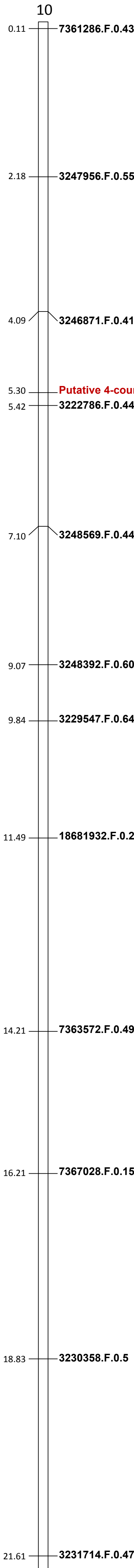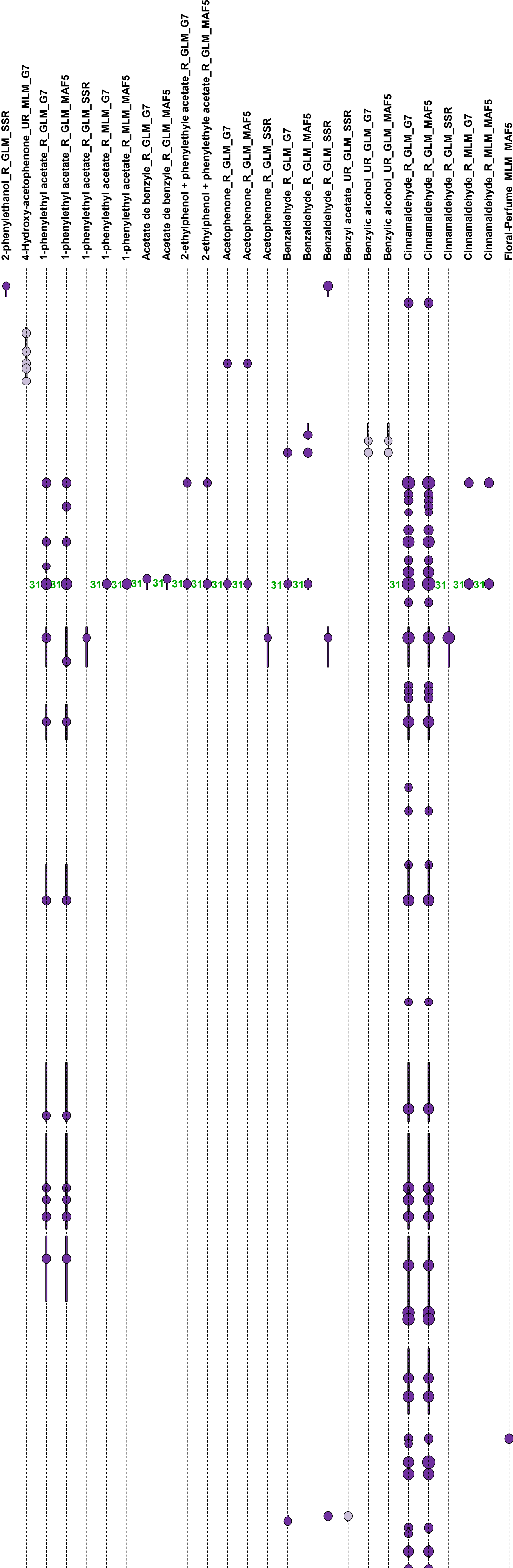

Supplement: Supplementary file 9 [file Image_6.PDF]

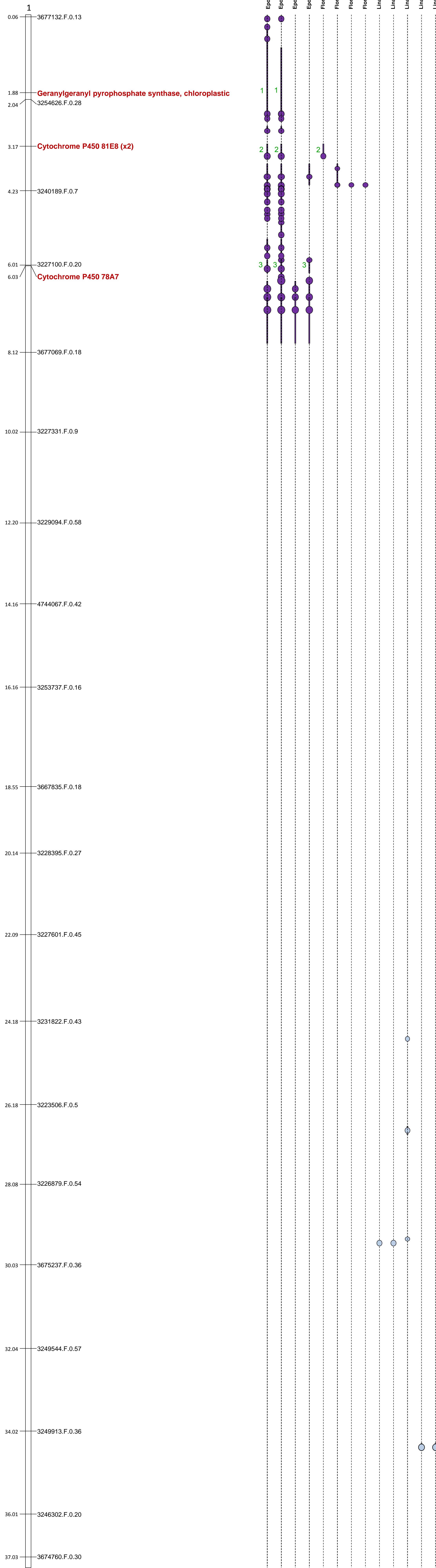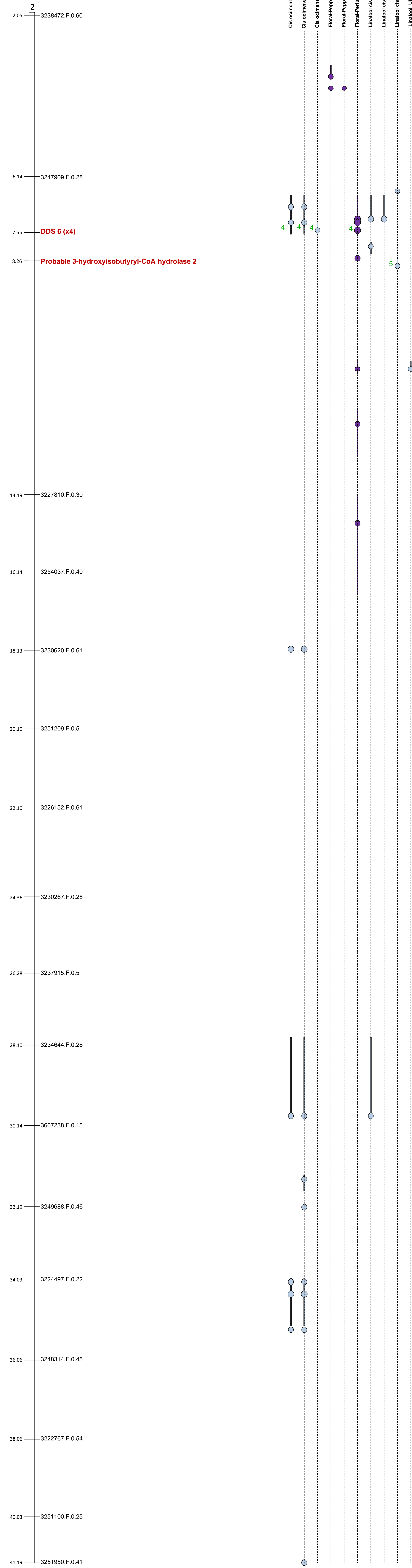

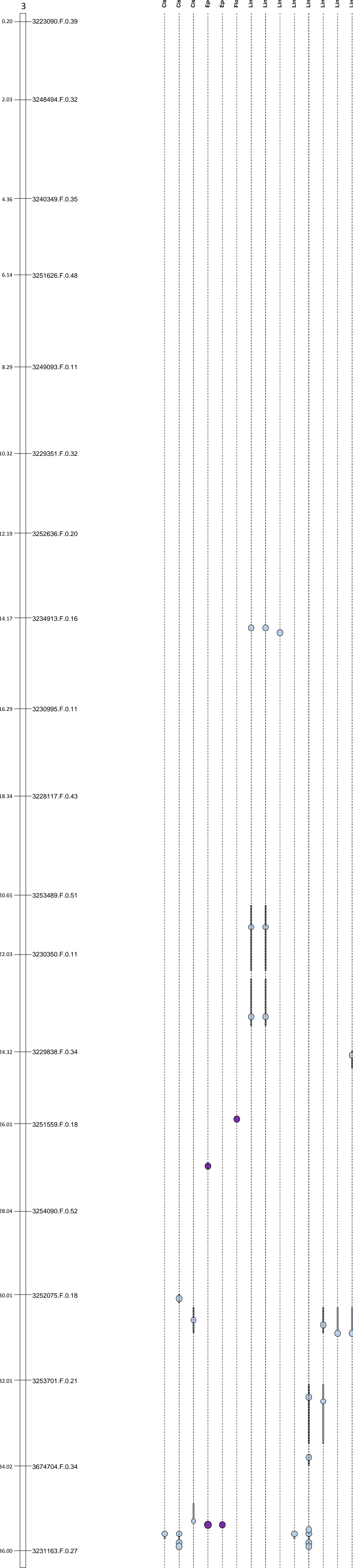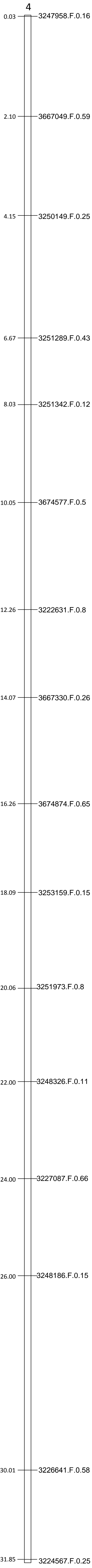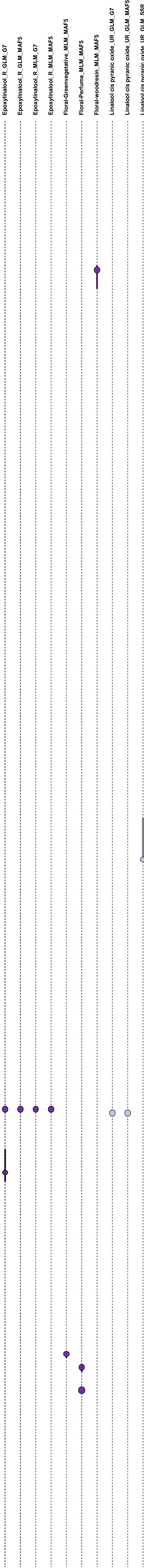

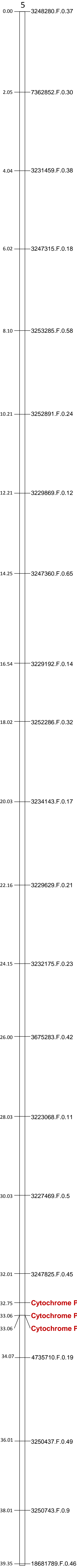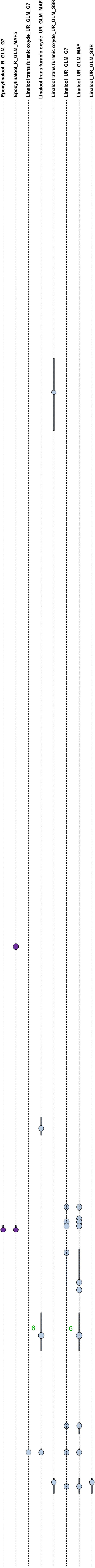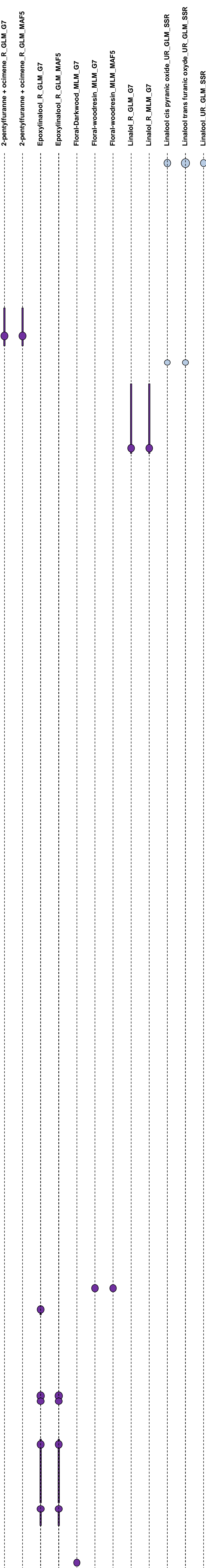

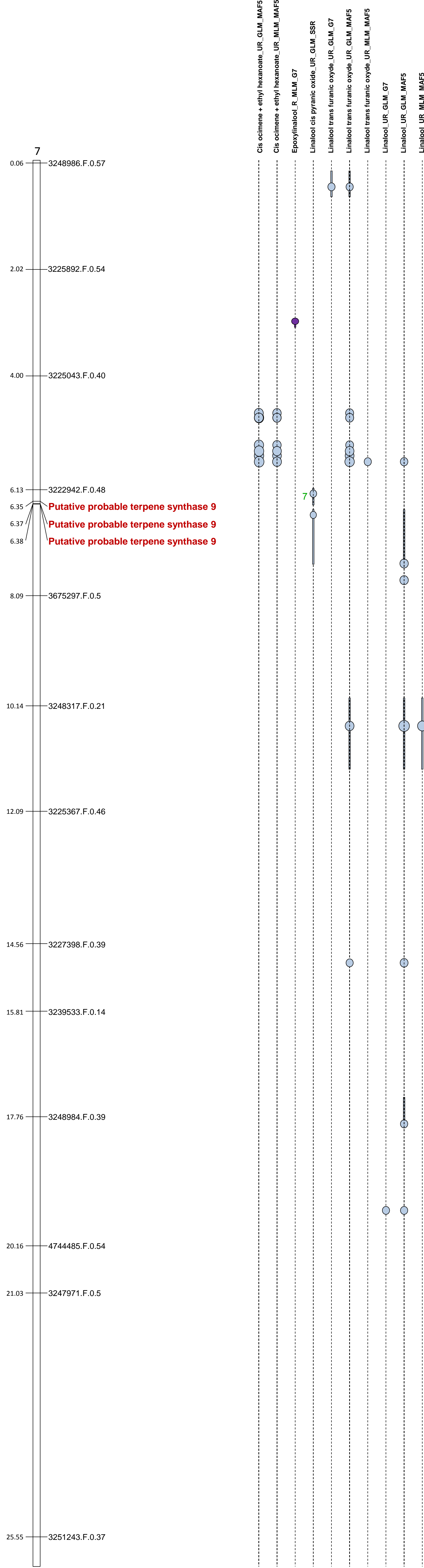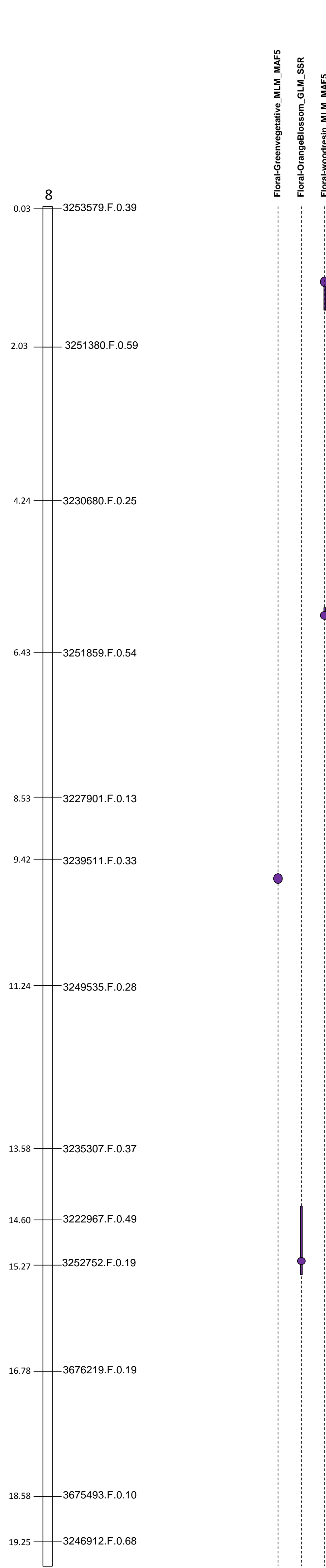

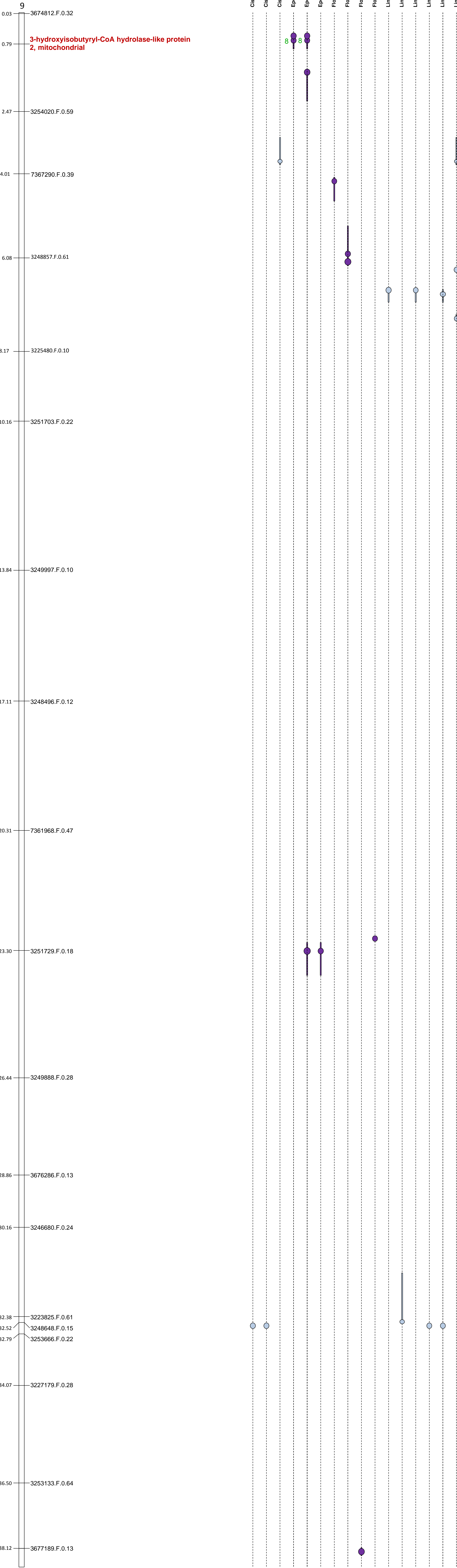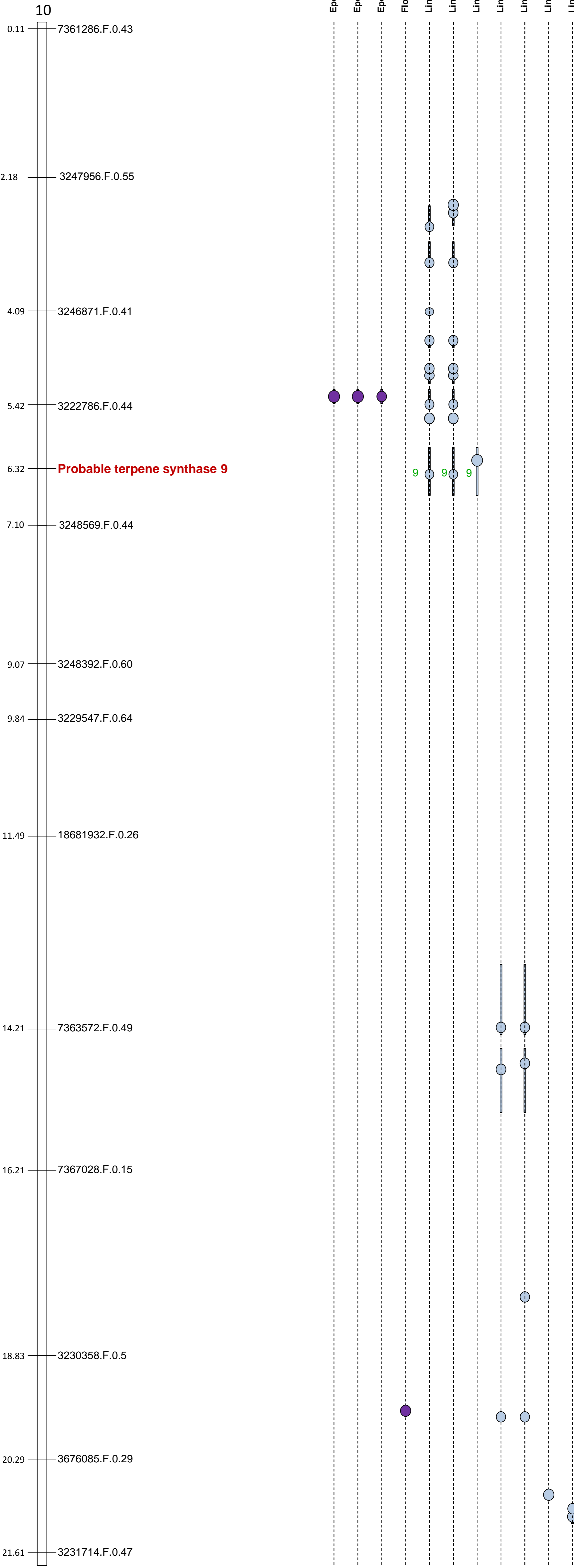

Supplement: Supplementary file 10 [file Image_7.PDF]
